# Integrated Evaluation of the Synergistic Antitumor Effects of Thymoquinone and Docetaxel in Ovarian Cancer Cells: Apoptosis, Oxidative Stress, and 3D Spheroid Responses

**DOI:** 10.3390/biomedicines14061341

**Published:** 2026-06-13

**Authors:** Aylin Orhaner, Mehmet Cudi Tuncer, İlhan Özdemir

**Affiliations:** 1 Department of Gynecology and Obstetrics, Medicana Bursa Hospital, 16150 Bursa, Turkey; draylinorhaner@hotmail.com; 2 Department of Anatomy, Faculty of Medicine, Dicle University, 21280 Diyarbakır, Turkey; 3 Department of Histology and Embryology, Faculty of Medicine, Kahramanmaraş Sütçü İmam University, 46000 Kahramanmaraş, Turkey; ilhanozdemir32@hotmail.com

**Keywords:** thymoquinone, docetaxel, OVCAR3, HaCaT, apoptosis, cell viability

## Abstract

**Background/Objectives**: The toxic side effects and resistance-associated limitations of conventional chemotherapeutic agents necessitate the development of more effective and selective combination strategies incorporating naturally derived compounds. In this study, the cytotoxic, apoptotic, oxidative stress-associated, and immunomodulatory effects of thymoquinone (TQ), a bioactive compound derived from *Nigella sativa*, and docetaxel (Dos), a taxane-based chemotherapeutic agent, were investigated alone and in combination in OVCAR3 ovarian cancer cells using integrated two-dimensional (2D) and three-dimensional (3D) experimental models. **Materials and Methods**: Cell viability was evaluated following treatment with TQ (10–500 µM), Dos (1–500 nM), and the TQ + Dos combination, and synergistic interactions were assessed by IC_50_^−^ and combination index-based analyses. Apoptosis and cell cycle distribution were analyzed by flow cytometry. Cytokine levels were determined using ELISA, whereas apoptosis- and cell cycle-associated gene expression profiles were evaluated by RT-qPCR. Active caspase-3 expression was assessed by immunocytochemistry. Intracellular reactive oxygen species (ROS) accumulation was examined using DCFH-DA-based fluorescence imaging and antioxidant rescue experiments using N-acetyl-L-cysteine (NAC). In addition, the antitumor activity of the combination was further evaluated in OVCAR3-derived 3D tumor spheroid models using spheroid morphology, ATP-based viability, and live/dead fluorescence imaging analyses. **Results**: The TQ + Dos combination demonstrated enhanced cytotoxic and apoptotic activity in OVCAR3 cells compared with single-agent treatments and induced marked G2/M cell cycle arrest. Combination treatment increased pro-apoptotic gene expression and was associated with reduced expression of anti-apoptotic markers and modulated inflammatory cytokine profiles. Fluorescence-based analyses demonstrated marked intracellular ROS accumulation following TQ + Dos treatment, whereas NAC pretreatment partially attenuated oxidative stress and restored viability, suggesting partial involvement of ROS-associated mechanisms in treatment-induced cytotoxicity. Importantly, the combination maintained stronger cytotoxic and growth-inhibitory effects than either monotherapy in 3D ovarian cancer spheroids, where combination treatment induced pronounced spheroid shrinkage, viability loss, and structural disruption. Relatively lower toxicity observed in HaCaT cells suggested partial selectivity toward cancer cells. **Conclusions**: Collectively, these in vitro findings suggest that the TQ + Dos combination produces greater cytotoxic, apoptotic, and growth-inhibitory effects than either agent alone in ovarian cancer models and is associated with alterations in apoptosis-, cell cycle-, and oxidative stress-related responses. The observation of these effects in 3D spheroid models supports further investigation of this combination in more advanced preclinical systems.

## 1. Introduction

Ovarian cancer remains one of the most aggressive and lethal gynecological malignancies worldwide and continues to represent a major public health burden due to its high mortality rate and frequent late-stage diagnosis [1,2]. Because early-stage disease is generally asymptomatic, most patients are diagnosed at advanced stages characterized by extensive peritoneal dissemination and chemoresistant tumor progression, resulting in poor long-term survival outcomes [3]. Despite substantial advances in surgical techniques and systemic treatment strategies, the standard therapeutic approach for ovarian cancer still primarily relies on cytoreductive surgery combined with platinum- and taxane-based chemotherapy [4]. Recent advances in ovarian cancer management have increasingly incorporated molecularly guided therapeutic strategies. In particular, BRCA1/2 mutation testing and assessment of homologous recombination deficiency (HRD) have become important components of clinical decision-making because of their predictive value for responsiveness to poly polymerase (PARP) inhibitor-based therapies. The introduction of PARP inhibitors has significantly improved progression-free survival in selected patient populations and has expanded the role of precision medicine in ovarian cancer treatment. Nevertheless, therapeutic resistance, disease recurrence, and treatment-associated toxicities remain major clinical challenges, highlighting the continued need for novel combination strategies capable of improving therapeutic efficacy while maintaining acceptable safety profiles. Among taxane derivatives, docetaxel (Dos) is widely used because of its potent antiproliferative activity mediated through stabilization of microtubules, disruption of mitotic spindle dynamics, induction of G2/M phase arrest, and subsequent activation of apoptotic pathways [5]. However, the clinical efficacy of docetaxel is frequently limited by cumulative toxicity, including hematological toxicity, peripheral neuropathy, hypersensitivity reactions, and the progressive emergence of chemoresistance during treatment [6]. These limitations have increased the need for alternative therapeutic strategies capable of enhancing antitumor efficacy while reducing systemic toxicity.

In recent years, combination approaches integrating conventional chemotherapeutic agents with naturally derived bioactive compounds have attracted considerable attention because of their potential to improve therapeutic selectivity, enhance treatment responsiveness, and modulate multiple cancer-associated signaling pathways simultaneously [7,8,9]. Naturally occurring phytochemicals are particularly attractive due to their relatively low toxicity profiles and their ability to regulate apoptosis, oxidative stress, inflammation, angiogenesis, and cell cycle progression. In this context, thymoquinone (TQ; 2-isopropyl-5-methylbenzo-1,4-quinone), the principal bioactive constituent of *Nigella sativa* L., has emerged as a promising anticancer compound with pleiotropic biological activities [10,11]. Previous studies demonstrated that TQ exerts antiproliferative, pro-apoptotic, anti-inflammatory, antioxidant, and antiangiogenic effects in various malignancies, including breast, colon, lung, pancreatic, and ovarian cancers [12,13,14]. Mechanistically, TQ has been associated with modulation of multiple signaling pathways involved in tumor progression and survival, including p53-associated signaling, the Bcl-2/Bax apoptotic axis, caspase activation cascades, NF-κB signaling, oxidative stress responses, and mitochondrial dysfunction [14,15]. In addition, accumulating evidence suggests that TQ may sensitize tumor cells to chemotherapeutic agents and thereby enhance treatment efficacy through multimodal molecular interactions [7,8,9].

The OVCAR3 ovarian adenocarcinoma cell line is widely used as an in vitro experimental model because it recapitulates several clinically relevant characteristics of high-grade ovarian cancer, including relative platinum resistance, aggressive growth behavior, and altered apoptotic signaling [16]. In contrast, the HaCaT immortalized human keratinocyte cell line serves as a non-tumoral reference model for evaluating the potential cytotoxic and selective effects of therapeutic agents on normal epithelial cells [17]. The combined use of OVCAR3 and HaCaT cells therefore provides a comparative framework for investigating both antitumor activity and treatment-associated toxicity.

Increasing evidence indicates that oxidative stress modulation and tumor microenvironment-associated responses may critically contribute to therapeutic sensitivity in ovarian cancer. Reactive oxygen species (ROS) are known to play dual roles in tumor biology by regulating proliferation, apoptosis, mitochondrial integrity, and inflammatory signaling pathways. Excessive ROS accumulation may promote cancer cell death, whereas antioxidant defense systems can contribute to treatment resistance. Accordingly, evaluating ROS-associated mechanisms together with antioxidant rescue approaches may provide mechanistic insight into treatment-induced cytotoxicity. Furthermore, although conventional two-dimensional (2D) monolayer cultures remain useful for mechanistic investigations, they incompletely reflect the structural complexity, diffusion gradients, and treatment resistance characteristics of solid tumors. Three-dimensional (3D) spheroid models more closely mimic in vivo tumor architecture and therefore represent valuable translational platforms for evaluating therapeutic responses under tumor-like conditions.

Although the potential anticancer activity of TQ and the therapeutic importance of taxane-based chemotherapy have been separately documented, the integrated molecular and functional effects of the TQ + Dos combination in ovarian cancer models remain insufficiently characterized, particularly with respect to oxidative stress-associated mechanisms, inflammatory modulation, and therapeutic activity in advanced 3D tumor models [18]. To the best of our knowledge, the combined effects of TQ and Dos have not been comprehensively investigated in OVCAR3 ovarian cancer cells using an integrated experimental framework incorporating apoptosis analysis, cell cycle profiling, cytokine evaluation, ROS modulation studies, active caspase-3 immunocytochemistry, three-dimensional spheroid validation, and complementary bioinformatic analyses. Therefore, a significant knowledge gap remains regarding the potential biological interactions between TQ and Dos and their collective impact on ovarian cancer-associated cellular responses. Therefore, the present study aimed to comprehensively investigate the cytotoxic, apoptotic, oxidative stress-associated, and immunomodulatory effects of TQ and Dos administered alone and in combination in OVCAR3 ovarian cancer cells while comparatively evaluating treatment-associated toxicity in HaCaT cells. To achieve this, integrated biological analyses including cell viability assays, flow cytometry, cytokine profiling, RT-qPCR, immunocytochemistry, fluorescence-based ROS imaging, antioxidant rescue experiments, and 3D spheroid analyses were performed. We hypothesized that the TQ + Dos combination would produce greater cytotoxic and apoptotic responses than either agent alone through modulation of apoptosis-, cell cycle-, and oxidative stress-associated pathways and that these responses could also be observed in 3D tumor-mimicking conditions.

## 2. Materials and Methods

### 2.1. Ovarian Cancer and Non-Tumoral Cell Culture Models

The human ovarian adenocarcinoma cell line OVCAR3 was obtained from the American Type Culture Collection (ATCC, Manassas, VA, USA), whereas the immortalized human keratinocyte cell line HaCaT was purchased from Cell Lines Service (CLS, Eppelheim, Germany). OVCAR3 cells were maintained in RPMI-1640 medium (Gibco, Thermo Fisher Scientific, Waltham, MA, USA) supplemented with 20% fetal bovine serum (FBS; Gibco, Thermo Fisher Scientific), 0.01 mg/mL insulin (Sigma-Aldrich, St. Louis, MO, USA), and 1% penicillin–streptomycin solution (Gibco, Thermo Fisher Scientific). HaCaT cells were cultured in Dulbecco’s Modified Eagle Medium (DMEM; Gibco, Thermo Fisher Scientific) supplemented with 10% FBS and 1% penicillin–streptomycin.

All cell cultures were maintained under standard humidified incubation conditions at 37 °C in an atmosphere containing 5% CO_2_. Culture media were refreshed every 2–3 days, and cells were routinely monitored microscopically for morphology and contamination status throughout the experimental period. Cells were subcultured upon reaching approximately 70–80% confluency using standard trypsinization procedures, and only cells with passage numbers below 15 were used in subsequent experiments to minimize phenotypic variation associated with prolonged culture adaptation.

### 2.2. Preparation of Thymoquinone and Docetaxel Treatment Conditions

Thymoquinone (TQ; purity ≥ 98%; Sigma-Aldrich, St. Louis, MO, USA) was dissolved in dimethyl sulfoxide (DMSO; Sigma-Aldrich) to prepare a 100 mM stock solution, whereas docetaxel (Dos; purity ≥ 95%; Sigma-Aldrich) was dissolved in absolute ethanol to obtain a 10 mM stock solution. Stock solutions were aliquoted and stored at −20 °C until use to minimize repeated freeze–thaw cycles. Prior to experimental applications, working concentrations were freshly prepared by dilution in the corresponding culture media. The final DMSO concentration was maintained below 0.1% in all experimental conditions, including vehicle controls, to avoid solvent-associated cytotoxicity.

For dose–response experiments, TQ was administered at concentrations of 10, 20, 50, 100, 250, and 500 µM, whereas Dos was applied at 1, 10, 50, 100, 250, and 500 nM. Cells were exposed to treatments for 24 and 48 h depending on the experimental design. For combination studies, a fixed-ratio treatment strategy was established based on the experimentally determined IC_50_ values of both agents in OVCAR3 cells. Combination treatments were administered simultaneously to evaluate potential synergistic interactions between TQ and Dos. Vehicle-treated control groups received equivalent concentrations of solvent under identical experimental conditions.

### 2.3. Evaluation of Cell Viability and Drug Combination Interactions

The effects of thymoquinone (TQ), docetaxel (Dos), and their combination on cellular viability were evaluated using the 3-(4,5-dimethylthiazol-2-yl)-2,5-diphenyltetrazolium bromide (MTT) assay. OVCAR3 and HaCaT cells were seeded into 96-well culture plates at a density of 5 × 10^3^ cells/well and allowed to attach for 24 h under standard incubation conditions prior to treatment exposure. Following attachment, cells were treated with increasing concentrations of TQ, Dos, or their combinations for 24 and 48 h.

At the end of the treatment period, 10 µL of MTT solution (5 mg/mL in phosphate-buffered saline (PBS); Sigma-Aldrich, St. Louis, MO, USA) was added to each well and incubated for an additional 4 h at 37 °C under light-protected conditions. Subsequently, the culture medium was carefully removed, and the resulting formazan crystals were dissolved in 100 µL dimethyl sulfoxide (DMSO). Absorbance values were measured at 570 nm using a microplate reader (BioTek, Winooski, VT, USA). Cell viability percentages were calculated relative to vehicle-treated control groups.

Dose–response curves and half-maximal inhibitory concentration (IC_50_) values were determined using a four-parameter nonlinear regression model in GraphPad Prism v9.0 software (GraphPad Software, San Diego, CA, USA). For combination experiments, synergistic interactions between TQ and Dos were evaluated using the Chou–Talalay method with CompuSyn software v1.0 (ComboSyn Inc., Paramus, NJ, USA). Combination index (CI) values were interpreted as follows: CI < 1 indicated synergism, CI = 1 indicated an additive effect, and CI > 1 indicated antagonism. IC_50_ values obtained from monotherapy experiments were used as operational reference concentrations for subsequent fixed-ratio combination treatments and mechanistic analyses. All experiments were independently repeated at least three times.

### 2.4. Flow Cytometric Evaluation of Apoptotic Cell Death

Apoptotic and necrotic cell death induced by TQ, Dos, and the TQ + Dos combination was evaluated using Annexin V-FITC/PI double staining followed by flow cytometric analysis. OVCAR3 cells were seeded into 6-well plates and treated with IC_50_-based concentrations of TQ, Dos, or TQ + Dos for 48 h. Following treatment, cells were harvested by trypsinization, washed twice with cold PBS, and resuspended in binding buffer at a concentration of 1 × 10^6^ cells/mL.

Subsequently, 5 µL Annexin V-FITC and 5 µL PI solution were added to each sample according to the manufacturer’s protocol (BD Biosciences, San Jose, CA, USA). Samples were incubated for 15 min at room temperature under light-protected conditions and immediately analyzed using a BD FACSCanto™ II flow cytometer (BD Biosciences, San Jose, CA, USA). At least 10,000 events were acquired for each sample.

Flow cytometric data were analyzed using FlowJo software v10.8 (BD Biosciences, San Jose, CA, USA). Cell populations were classified as viable, early apoptotic, late apoptotic, and necrotic according to Annexin V/PI staining profiles. Quantitative apoptotic distributions were calculated from three independent experiments.

### 2.5. Flow Cytometric Analysis of Cell Cycle Distribution

The effects of TQ, Dos, and the TQ + Dos combination on cell cycle progression were evaluated by PI-based DNA content analysis using flow cytometry. OVCAR3 cells were treated with IC_50_-based concentrations of TQ, Dos, or TQ + Dos for 48 h. Following treatment, cells were harvested, washed with PBS, and fixed in ice-cold 70% ethanol at 4 °C for 24 h.

After fixation, cells were washed twice with PBS and incubated in staining solution containing 50 µg/mL PI and 100 µg/mL RNase A (Sigma-Aldrich, St. Louis, MO, USA) for 30 min at 37 °C under light-protected conditions. Flow cytometric acquisition was performed using a BD FACSCanto™ II flow cytometer (BD Biosciences, San Jose, CA, USA), and at least 10,000 events were collected for each sample.

Cell cycle distributions corresponding to G0/G1, S, and G2/M phases were analyzed using ModFit LT software v5.0 (Verity Software House, Topsham, ME, USA). Quantitative phase distributions were calculated from three independent experiments.

### 2.6. Quantitative Analysis of Cytokine Profiles

To evaluate treatment-associated inflammatory responses, cytokine levels in cell culture supernatants were quantified using an enzyme-linked immunosorbent assay (ELISA). OVCAR3 cells were treated with IC_50_-based concentrations of TQ, Dos, or the TQ + Dos combination for 48 h, and culture supernatants were collected following centrifugation to remove cellular debris.

The concentrations of IL-6, IL-8, TNF-α, and IL-10 were determined using commercially available ELISA kits (R&D Systems, Minneapolis, MN, USA) according to the manufacturer’s protocols. Optical density values were measured at 450 nm using a microplate reader (BioTek, Winooski, VT, USA). Cytokine concentrations were calculated from standard calibration curves and expressed as pg/mL. All measurements were performed in triplicate from three independent experiments.

### 2.7. RNA Isolation and Complementary DNA (cDNA) Synthesis

Total RNA was isolated from OVCAR3 cells following TQ, Dos, and TQ + Dos treatments using TRIzol™ Reagent (Thermo Fisher Scientific, Waltham, MA, USA) according to the manufacturer’s instructions. Briefly, cells were lysed directly in TRIzol reagent, and RNA was separated by chloroform extraction followed by isopropanol precipitation. The resulting RNA pellets were washed with 75% ethanol, air-dried, and dissolved in RNase-free water.

RNA concentration and purity were evaluated using a NanoDrop™ 2000 spectrophotometer (Thermo Fisher Scientific) based on absorbance measurements at 260 and 280 nm. Only RNA samples with appropriate purity ratios were used for downstream analyses.

Complementary DNA (cDNA) synthesis was performed using 1 µg total RNA and the RevertAid First Strand cDNA Synthesis Kit (Thermo Fisher Scientific) according to the manufacturer’s protocol. Reverse transcription reactions were carried out at 42 °C for 60 min followed by enzyme inactivation according to kit instructions. Synthesized cDNA samples were stored at −20 °C until RT-qPCR analyses.

### 2.8. Quantitative Real-Time PCR (RT-qPCR) Analysis

The relative expression levels of apoptosis- and cell cycle-associated genes were evaluated by SYBR Green-based quantitative real-time PCR (RT-qPCR). OVCAR3 cells were treated with IC_50_-based concentrations of TQ, Dos, or the TQ + Dos combination for 48 h prior to RNA isolation and cDNA synthesis.

RT-qPCR reactions were performed using an Applied Biosystems™ 7500 Fast Real-Time PCR System (Thermo Fisher Scientific, Waltham, MA, USA). Amplification reactions were carried out using SYBR Green Master Mix under the following cycling conditions: initial denaturation at 95 °C for 10 min, followed by 40 amplification cycles consisting of denaturation at 95 °C for 15 s and annealing/extension at 60 °C for 1 min.

The expression levels of *BCL2*, *BAX*, *CASP3*, *CASP9*, *TP53*, *CDKN1A*, and *CDK4* were evaluated. *GAPDH* and *ACTB* were used as internal reference genes for normalization. Relative gene expression levels were calculated using the 2^−ΔΔCt^ method. Primer sequences used for RT-qPCR analyses are presented in Table 1. All reactions were performed in triplicate from three independent experiments.

### 2.9. Immunocytochemical Evaluation of Active Caspase-3 Expression

Immunocytochemical staining was performed to evaluate active caspase-3 protein expression following TQ, Dos, and TQ + Dos treatments in OVCAR3 cells. Cells were seeded onto sterile glass coverslips placed in 24-well plates and treated with IC_50_-based concentrations of TQ, Dos, or TQ + Dos for 48 h. Following treatment, cells were fixed with 4% paraformaldehyde (Sigma-Aldrich, St. Louis, MO, USA) for 15 min at room temperature.

Endogenous peroxidase activity was blocked using methanol containing 3% H_2_O_2_ for 10 min. Cells were subsequently permeabilized with PBS containing 0.3% Triton™ X-100 (Sigma-Aldrich) for 10 min and blocked with 5% normal goat serum for 1 h at room temperature to minimize nonspecific antibody binding.

Active caspase-3 expression was detected using a rabbit anti-active caspase-3 primary antibody (1:200 dilution; ab184787, Abcam, Cambridge, UK). Cells were incubated with the primary antibody overnight at 4 °C in a humidified chamber. Following PBS washing steps, a biotinylated secondary antibody (anti-rabbit IgG; 1:500 dilution) was applied for 30 min at room temperature, followed by incubation with streptavidin–horseradish peroxidase complex for an additional 30 min.

Immunoreactivity was visualized using a DAB chromogen substrate kit (Sigma-Aldrich). Color development was monitored microscopically and stopped by washing with distilled water once brown cytoplasmic staining became evident. Counterstaining was performed using Mayer’s hematoxylin, followed by dehydration through graded ethanol series, xylene clearing, and coverslipping with Entellan mounting medium.

Stained preparations were examined under a light microscope (Olympus, Tokyo, Japan). Semi-quantitative evaluation of active caspase-3 immunoreactivity was performed using the H-score method based on staining intensity and positively stained cell distribution.

### 2.10. Fluorescence-Based Visualization of Intracellular ROS Accumulation

Intracellular reactive oxygen species (ROS) accumulation was visualized using the fluorescent probe 2′,7′-dichlorodihydrofluorescein diacetate (DCFH-DA; Sigma-Aldrich, St. Louis, MO, USA). OVCAR3 cells were seeded into 6-well plates and cultured overnight under standard incubation conditions at 37 °C in a humidified atmosphere containing 5% CO_2_. Cells were treated with thymoquinone (TQ), docetaxel (Dos), or the TQ + Dos combination for 48 h using previously determined IC50 concentrations. For antioxidant rescue experiments, cells were pretreated with N-acetyl-L-cysteine (NAC, 5 mM) for 1 h prior to treatment exposure.

Following treatment, cells were washed twice with phosphate-buffered saline (PBS) and incubated with DCFH-DA solution (10 µM) for 30 min at 37 °C under light-protected conditions. After staining, excess dye was removed by washing with PBS. Representative fluorescence captures were obtained using an inverted fluorescence microscope (Olympus, Tokyo, Japan) equipped with appropriate excitation and emission filters. All fluorescence images were acquired using identical exposure time, gain, and acquisition settings across experimental groups to ensure direct comparability. Green fluorescence intensity was interpreted as an indicator of intracellular ROS accumulation.

### 2.11. Three-Dimensional (3D) Tumor Spheroid Modeling and Functional Evaluation of the TQ + Dos Combination

To better mimic the architectural organization, diffusion barriers, and microenvironmental complexity of solid ovarian tumors, a three-dimensional (3D) tumor spheroid model was established using OVCAR3 cells.

#### 2.11.1. Generation of Uniform OVCAR3 Tumor Spheroids

OVCAR3 cells were cultured in RPMI-1640 medium supplemented with 20% fetal bovine serum (FBS; Gibco, Thermo Fisher Scientific, Waltham, MA, USA), 1% penicillin–streptomycin, and 0.01 mg/mL insulin under standard humidified culture conditions at 37 °C and 5% CO_2_. Cells in the logarithmic growth phase were seeded into ultra-low attachment round-bottom 96-well plates (Corning Inc., Corning, NY, USA) at a density of 3 × 10^3^ cells/well in a final volume of 200 µL. Plates were incubated under standard conditions to allow spontaneous spheroid formation. Compact and morphologically homogeneous spheroids formed within 48–72 h and were selected for subsequent analyses.

#### 2.11.2. Treatment of 3D Tumor Spheroids

Following spheroid maturation, culture medium was carefully replaced with fresh medium containing thymoquinone (TQ), docetaxel (Dos), or the TQ + Dos combination. Drug concentrations were selected according to IC_50_ values obtained from two-dimensional monolayer experiments and were used as operational reference concentrations for 3D studies. Vehicle-treated spheroids served as controls. Due to reduced proliferation rates and diffusion limitations characteristic of 3D structures, spheroids were exposed to treatments for 72 h under standard culture conditions.

#### 2.11.3. Bright-Field Imaging and Morphological Evaluation

Morphological alterations in spheroids were documented using an inverted bright-field microscope (Olympus, Tokyo, Japan). Images were captured using identical optical settings across all groups to ensure direct comparability. Spheroid morphology was qualitatively assessed based on compactness, structural integrity, cellular dispersion, peripheral loosening, and fragmentation.

#### 2.11.4. Quantitative Measurement of Spheroid Diameter

Spheroid diameter was quantified using ImageJ software (version 1.53; National Institutes of Health, Bethesda, MD, USA). For each spheroid, two perpendicular diameter measurements were obtained at the widest regions, and the mean value was calculated. Analyses were performed using spheroids derived from at least three independent biological replicates.

#### 2.11.5. ATP-Based Analysis of 3D Spheroid Viability

Cell viability within spheroids was evaluated using a luminescence-based ATP assay optimized for three-dimensional cultures (CellTiter-Glo^®^ 3D Cell Viability Assay; Promega, Madison, WI, USA). Equal volumes of reagent were added directly to each well to ensure complete spheroid lysis and ATP release. Following incubation for signal stabilization, luminescence intensity was measured using a microplate reader (BioTek Instruments, Winooski, VT, USA). Viability values were normalized to vehicle-treated controls and expressed as percentages.

#### 2.11.6. Live/Dead Fluorescence Imaging of 3D Tumor Spheroids

Spatial treatment-associated cytotoxicity within spheroids was evaluated using fluorescence-based live/dead staining with Calcein-AM and Ethidium homodimer-1 (Thermo Fisher Scientific, Waltham, MA, USA). Following staining, spheroids were visualized using an inverted fluorescence microscope (Olympus, Tokyo, Japan) equipped with appropriate excitation and emission filters. Images were acquired using identical exposure settings and focal planes for all groups without post-acquisition image manipulation. Green fluorescence represented viable cells, whereas red fluorescence indicated membrane-compromised dead cells. Live/dead staining patterns were interpreted together with spheroid morphology to evaluate treatment-associated structural disruption and cell death distribution within the 3D tumor architecture.

### 2.12. Functional Evaluation of ROS Contribution Using NAC-Mediated Antioxidant Rescue

To investigate the contribution of reactive oxygen species (ROS) to TQ + Dos-associated cytotoxicity, an antioxidant rescue approach using N-acetyl-L-cysteine (NAC) was performed in the 3D spheroid model.

#### 2.12.1. NAC Pretreatment and Experimental Design

Mature and morphologically uniform OVCAR3 spheroids were pretreated with NAC (5 mM; Sigma-Aldrich, St. Louis, MO, USA) for 1 h at 37 °C in a humidified 5% CO_2_ atmosphere prior to treatment exposure. This concentration was selected to ensure effective intracellular ROS scavenging while minimizing nonspecific cytotoxicity. Following pretreatment, spheroids were treated with TQ, Dos, or the TQ + Dos combination using concentrations identical to those applied in the 3D spheroid experiments. Untreated spheroids and NAC-only treated spheroids were included as controls.

#### 2.12.2. Quantification of Intracellular ROS Levels

Intracellular ROS levels were quantified using the fluorescent probe DCFH-DA (Sigma-Aldrich, St. Louis, MO, USA). Following treatment, spheroids were enzymatically dissociated into single-cell suspensions using Accutase^®^ (Sigma-Aldrich) to enable quantitative fluorescence analysis. Cells were incubated with DCFH-DA (10 µM) for 30 min at 37 °C under light-protected conditions. After washing with PBS, fluorescence intensity was measured using a microplate reader at excitation/emission wavelengths of 485/535 nm. ROS levels were expressed as fold change relative to untreated controls.

#### 2.12.3. Assessment of Cytotoxicity Following ROS Neutralization

The effect of ROS scavenging on treatment-associated cytotoxicity was evaluated using the CellTiter-Glo^®^ 3D ATP-based luminescence assay. Equal volumes of reagent were added directly to spheroids to ensure complete lysis and ATP release. Luminescence intensity was measured using a microplate reader, and viability values were normalized to untreated controls. Partial restoration of viability following NAC pretreatment was interpreted as functional evidence supporting ROS involvement in TQ + Dos-mediated cytotoxicity.

### 2.13. Bioinformatics Analysis

To explore the potential molecular mechanisms associated with TQ and Dos treatment, an integrated bioinformatics analysis workflow was performed using publicly accessible pharmacological and disease-related databases. Putative molecular targets of TQ were identified using the SwissTargetPrediction (http://www.swisstargetprediction.ch; accessed on 16 April 2026) and PharmMapper (http://www.lilab-ecust.cn/pharmmapper/; accessed on 16 April 2026) databases based on chemical structure-associated target prediction approaches. Known pharmacological targets associated with Dos were retrieved from the DrugBank database (https://go.drugbank.com; accessed on 16 April 2026).

Ovarian cancer-associated genes were collected from the GeneCards (https://www.genecards.org; accessed on 16 April 2026) and DisGeNET (https://www.disgenet.org; accessed on 16 April 2026) databases using the keyword “ovarian cancer”. To improve disease relevance and reduce low-confidence associations, only genes meeting a relevance score threshold of ≥0.1 were included in subsequent analyses.

Overlapping genes between drug-associated targets and ovarian cancer-related genes were identified to determine potential therapeutic target candidates associated with the TQ + Dos combination. These overlapping targets were subsequently used for downstream enrichment and interaction analyses to evaluate potential biological processes and signaling pathways associated with treatment responses. Bioinformatics findings were interpreted as hypothesis-generating computational predictions intended to support experimental observations rather than establish direct mechanistic causality.

#### 2.13.1. Protein–Protein Interaction (PPI)

To evaluate potential functional interactions among overlapping therapeutic target genes, a protein–protein interaction (PPI) network was constructed using the STRING database v11.5 (https://string-db.org; accessed on 16 April 2026). The minimum required interaction score was set to 0.7 to ensure high-confidence interaction prediction. Isolated nodes without significant interactions were excluded from the final network analysis.

The resulting interaction network was imported into Cytoscape software v3.10.0 (https://cytoscape.org; accessed on 16 April 2026) for network visualization and topological analysis. Network parameters, including node degree and interaction connectivity, were evaluated to identify potential hub genes associated with the TQ + Dos treatment network. The generated PPI network was used as a computational framework to support downstream enrichment analyses and interpretation of experimentally observed molecular responses.

#### 2.13.2. Gene Ontology (GO) and KEGG Pathway Enrichment Analysis

Functional enrichment analyses were performed to explore biological processes and pathways associated with overlapping target genes associated with the TQ + Dos treatment network. Gene Ontology (GO) analysis was conducted to classify enriched targets according to biological processes, molecular functions, and cellular components, whereas Kyoto Encyclopedia of Genes and Genomes (KEGG) pathway enrichment analysis was performed to identify significantly associated signaling pathways and cancer-related molecular networks.

Enrichment analyses and data visualization were performed using appropriate bioinformatics platforms and integrated computational tools. To minimize false-positive enrichment bias, multiple testing correction was applied using the Benjamini–Hochberg method. GO terms and KEGG pathways with a false discovery rate (FDR) < 0.05 and a minimum gene count of ≥5 were considered statistically significant.

The identified enriched pathways and biological processes were interpreted as computationally predicted associations intended to support experimental findings and provide mechanistic insight into potential molecular responses associated with TQ + Dos treatment.

### 2.14. Statistical Analysis

All quantitative experiments were independently repeated in three biological replicates (*n* = 3), and quantitative data are presented as mean ± standard deviation (SD). Statistical analyses were performed using GraphPad Prism v9.0 software (GraphPad Software, San Diego, CA, USA).

Comparisons among multiple experimental groups were performed using one-way analysis of variance (ANOVA) followed by Tukey’s post hoc multiple comparison test. Comparisons between two groups were evaluated using Student’s *t*-test where appropriate. A *p*-value < 0.05 was considered statistically significant.

For RT-qPCR analyses, relative gene expression levels were calculated using the 2^−ΔΔCt^ method prior to statistical evaluation. Flow cytometry distributions, cytokine measurements, fluorescence intensity analyses, spheroid diameter measurements, ATP-based viability analyses, and immunocytochemical H-score evaluations were analyzed using the same statistical workflow.

Combination index (CI) values generated using the Chou–Talalay method were interpreted descriptively to assess potential synergistic interactions between TQ and Dos and were not subjected to inferential statistical comparison.

## 3. Results

### 3.1. Effects of TQ, Dos, and Their Combination on Cell Viability

**Figure 1:**
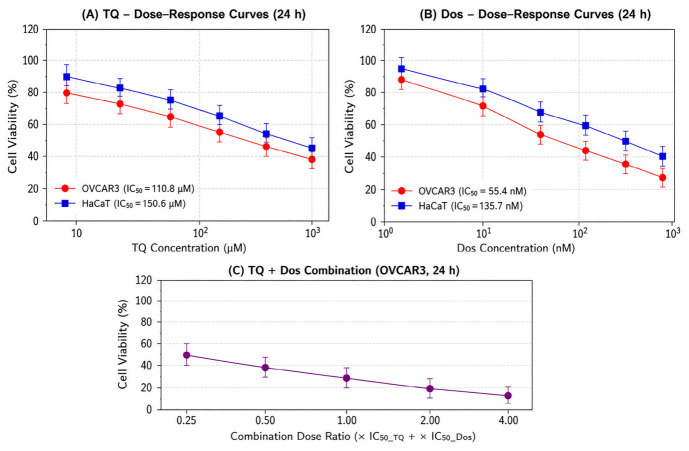
Effects of TQ, Dos, and their combination on cell viability in OVCAR3 and HaCaT cells after 24 h treatment. (**A**) Dose–response curves of TQ in OVCAR3 and HaCaT cells following 24 h exposure. (**B**) Dose–response curves of Dos in OVCAR3 and HaCaT cells following 24 h exposure. IC_50_ values are indicated within the graphs. (**C**) Cell viability of OVCAR3 cells treated with fixed-ratio combinations of TQ and Dos based on their respective IC_50_ values. Cell viability was determined using the MTT assay. Data are presented as mean ± SD (*n* = 3) from three independent biological experiments.

The cytotoxic effects of TQ, Dos, and the TQ + Dos combination on OVCAR3 and HaCaT cells were evaluated using the MTT assay following 24 h of treatment (Figure 1). TQ treatment reduced cell viability in both cell lines in a concentration-dependent manner, with a more pronounced reduction observed in OVCAR3 cells compared with HaCaT cells (Figure 1A). The IC_50_ value of TQ at 24 h was calculated as 110.8 µM in OVCAR3 cells and 150.6 µM in HaCaT cells, indicating relatively greater sensitivity of ovarian cancer cells to TQ treatment.

Similarly, Dos treatment induced dose-dependent cytotoxicity in both cell lines, although OVCAR3 cells exhibited substantially higher sensitivity than HaCaT cells (Figure 1B). The IC_50_ value of Dos was determined as 55.4 nM in OVCAR3 cells and 135.7 nM in HaCaT cells after 24 h of exposure, supporting the selective antitumor activity of Dos against ovarian cancer cells.

Combination treatment with TQ + Dos produced a markedly greater reduction in OVCAR3 cell viability compared with either monotherapy alone (Figure 1C). Progressive decreases in viability were observed with increasing combination dose ratios, reaching approximately 15–20% viability at the highest tested combination concentration. These findings suggest enhanced cytotoxic activity of the combined treatment relative to single-agent exposure.

Overall, both TQ and Dos demonstrated greater cytotoxic activity in OVCAR3 cells than in non-tumoral HaCaT cells, whereas the TQ + Dos combination further potentiated the reduction in ovarian cancer cell viability. The observed decrease in IC_50_ values and enhanced suppression of cell survival following combination treatment are consistent with a potential synergistic interaction between TQ and Dos.

**Figure 2:**
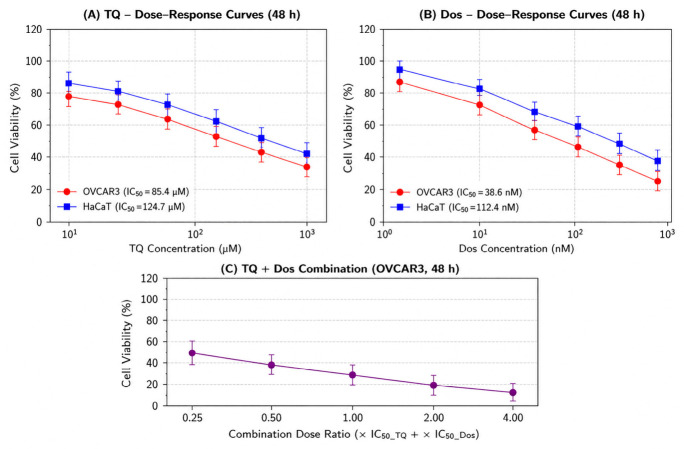
Effects of TQ, Dos, and their combination on cell viability in OVCAR3 and HaCaT cells after 48 h treatment. (**A**) Dose–response curves of TQ in OVCAR3 and HaCaT cells following 48 h exposure. (**B**) Dose–response curves of Dos in OVCAR3 and HaCaT cells following 48 h exposure. IC_50_ values are indicated within the graphs. (**C**) Cell viability of OVCAR3 cells treated with fixed-ratio combinations of TQ and Dos based on their respective IC_50_ values. Cell viability was determined using the MTT assay. Data are presented as mean ± SD (*n* = 3) from three independent biological experiments.

Following 48 h exposure, both TQ and Dos produced more pronounced cytotoxic effects in OVCAR3 and HaCaT cells compared with the 24 h treatment period (Figure 2). TQ treatment resulted in a concentration-dependent reduction in cell viability in both cell lines, with OVCAR3 cells exhibiting greater sensitivity than HaCaT cells (Figure 2A). The IC_50_ value of TQ decreased from 110.8 µM at 24 h to 85.4 µM at 48 h in OVCAR3 cells, whereas the IC_50_ value in HaCaT cells decreased to 124.7 µM, indicating time-dependent enhancement of TQ-associated cytotoxicity. Similarly, Dos treatment induced a marked reduction in cell viability after 48 h exposure (Figure 2B). The IC_50_ value of Dos was calculated as 38.6 nM in OVCAR3 cells and 112.4 nM in HaCaT cells, further supporting the greater susceptibility of ovarian cancer cells to Dos treatment relative to non-tumoral cells. The TQ + Dos combination exhibited the strongest cytotoxic activity among all treatment conditions (Figure 2C). Increasing combination dose ratios progressively reduced OVCAR3 cell viability, reaching approximately 15% viability at the highest tested concentration. Compared with monotherapy treatments, the combination induced a substantially greater reduction in cell survival, suggesting enhanced antitumor activity following combined exposure. Overall, the 48 h treatment period resulted in lower viability values and reduced IC_50_ concentrations compared with 24 h exposure, indicating a time-dependent increase in treatment efficacy for both TQ and Dos.

### 3.2. Synergistic Cytotoxic Interaction Between TQ and Dos

**Figure 3:**
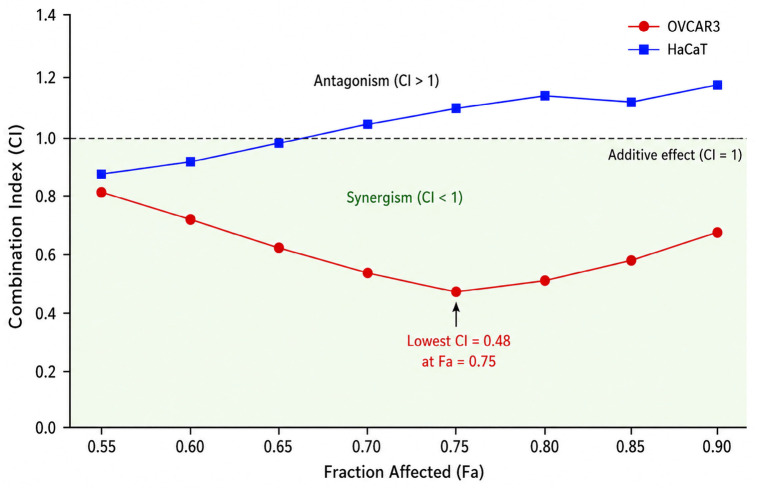
Synergistic interaction analysis of the TQ + Dos combination in OVCAR3 and HaCaT cells based on the Chou–Talalay method. CI values were plotted against the Fa following treatment with fixed-ratio combinations of TQ and Dos. In OVCAR3 cells, CI values remained below 1.0 across all evaluated effect levels (Fa = 0.55–0.90), indicating synergistic interactions throughout the tested concentration range. The strongest synergistic effect was observed at Fa = 0.75, where the lowest CI value (0.48) was obtained. In contrast, HaCaT cells exhibited CI values ranging from 0.88 to 1.18, indicating limited synergistic or predominantly additive interactions in non-tumoral cells. The dashed horizontal line represents the additive threshold (CI = 1), while the shaded region indicates synergistic interactions (CI < 1). CI values were calculated from MTT viability data obtained after 48 h treatment (*n* = 3 independent biological experiments).

**Figure 4:**
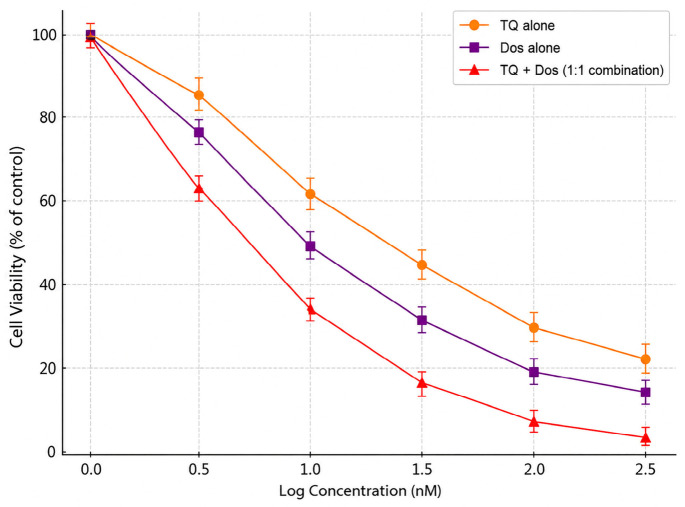
Dose–response analysis of TQ, Dos, and the TQ + Dos combination in OVCAR3 cells following 48 h treatment. Cell viability was assessed using the MTT assay after exposure to increasing concentrations of TQ, Dos, or their fixed-ratio combination. The combination treatment produced a greater reduction in cell viability than either single-agent treatment across the evaluated concentration range. Data are presented as mean ± SD (*n* = 3) from three independent biological experiments.

The potential synergistic interaction between TQ and Dos was evaluated using fixed-ratio combination treatment and analyzed according to the Chou–Talalay method. Combination treatment produced a markedly greater reduction in OVCAR3 cell viability compared with either monotherapy alone (Figure 3 and Figure 4). CI–Fa analysis demonstrated that CI values in OVCAR3 cells remained below 1.0 across all evaluated effect fractions (Fa = 0.55–0.90), indicating synergistic interaction throughout the tested concentration range (Figure 3). The strongest synergistic interaction was observed at Fa = 0.75, where the lowest CI value was calculated as 0.48.

In contrast, HaCaT cells exhibited substantially weaker synergistic responses following combination treatment. CI values in HaCaT cells ranged between 0.88 and 1.18, indicating limited synergy and partial additive effects in non-tumoral cells. These findings suggest that the synergistic cytotoxic activity of the TQ + Dos combination was more pronounced in ovarian cancer cells than in normal keratinocyte cells.

Dose–response curve analysis further supported the synergistic interaction between TQ and Dos in OVCAR3 cells (Figure 4). Compared with TQ or Dos monotherapy, the TQ + Dos combination shifted the dose–response curve leftward and produced a more substantial reduction in cell viability at corresponding concentration levels. At higher treatment concentrations, combination therapy reduced cell viability to below 10%, whereas monotherapy groups retained relatively higher viability levels.

Overall, these findings indicate that combined TQ and Dos treatment exerts enhanced cytotoxic activity in OVCAR3 cells and supports a potential synergistic interaction between the two agents under the tested experimental conditions.

### 3.3. Induction of Apoptosis Following TQ and Dos Treatment

**Figure 5:**
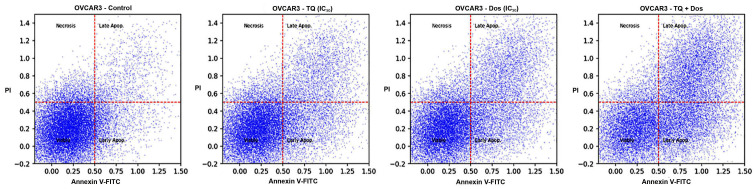
Flow cytometric analysis of apoptosis in OVCAR3 cells following 48 h treatment with TQ, Dos, and the TQ + Dos combination. Representative Annexin V-FITC/PI dot plots demonstrate the distribution of viable, early apoptotic, late apoptotic, and necrotic cell populations. Control cells were predominantly localized within the viable quadrant, whereas TQ and Dos treatment increased Annexin V-positive cell populations. The TQ + Dos combination produced the strongest apoptotic response, characterized by marked accumulation of cells within the apoptotic quadrants and a substantial reduction in viable cell density. Quadrants indicate viable cells (Annexin V^−^/PI^−^), early apoptotic cells (Annexin V^+^/PI^−^), late apoptotic cells (Annexin V^+^/PI^+^), and necrotic cells (Annexin V^−^/PI^+^). Data are representative of three independent biological experiments (*n* = 3).

Apoptotic alterations induced by TQ, Dos, and their combination were evaluated in OVCAR3 cells using Annexin V-FITC/PI staining after 48 h treatment (Figure 5). Flow cytometric dot plot analysis demonstrated clear treatment-associated shifts in cell population distribution compared with the control group.

The control group mainly consisted of viable cells localized within the Annexin V^−^/PI^−^ quadrant, with minimal apoptotic distribution. TQ treatment increased the proportion of Annexin V-positive cells, indicating induction of apoptotic cell death. A more evident apoptotic shift was observed following Dos treatment, with increased accumulation of cells in both early and late apoptotic quadrants.

Among all experimental conditions, the TQ + Dos combination produced the most pronounced apoptotic profile. Combination-treated cells exhibited marked expansion of the Annexin V-positive populations together with a substantial decline in viable cells. In particular, increased density within the late apoptotic quadrant suggested enhanced progression toward irreversible apoptotic damage following combined exposure.

These findings indicate that co-treatment with TQ and Dos intensified apoptosis-related cellular responses in OVCAR3 cells compared with either monotherapy alone.

### 3.4. Effects of TQ and Dos on Cell Cycle Distribution

**Figure 6:**
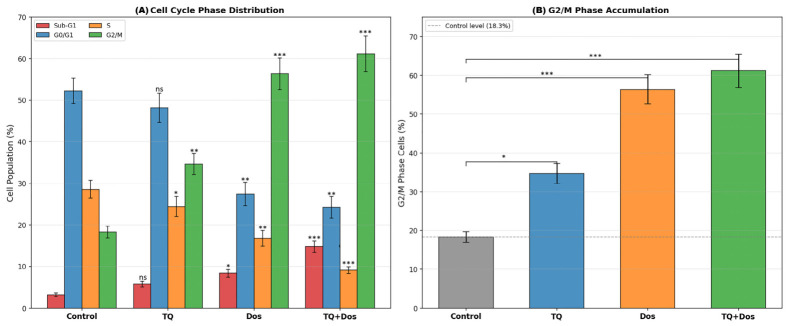
Cell cycle distribution analysis in OVCAR3 cells following 48 h treatment with TQ, Dos, and the TQ + Dos combination. (**A**) Distribution of cell populations within sub-G1, G0/G1, S, and G2/M phases. Treatment with TQ and Dos increased the proportion of cells in the G2/M phase relative to the control group, whereas combination treatment produced the most pronounced G2/M accumulation together with a marked reduction in S-phase cells. The TQ + Dos group also demonstrated increased sub-G1 population levels, indicating apoptosis-associated DNA fragmentation. (**B**) Quantitative comparison of G2/M phase accumulation among treatment groups. The proportion of G2/M phase cells increased from 18.3% in control cells to 34.8% following TQ treatment, 56.4% following Dos treatment, and 61.2% following combination treatment. Data are presented as mean ± SD (*n* = 3) from three independent biological experiments (* *p* < 0.05, ** *p* < 0.01, *** *p* < 0.001 versus control, ns, not significant).

The effects of TQ, Dos, and combination treatment on cell cycle progression in OVCAR3 cells were evaluated by flow cytometric analysis following 48 h exposure (Figure 6). Distinct alterations in phase distribution were observed among treatment groups compared with the control condition.

Control cells predominantly accumulated within the G0/G1 phase (52.4 ± 3.1%), whereas the G2/M population remained comparatively low (18.3 ± 1.4%). TQ treatment moderately increased G2/M phase accumulation to 34.8 ± 2.9% (*p* < 0.05), accompanied by a reduction in the G0/G1 population. Dos treatment produced a substantially stronger effect on cell cycle progression, increasing the G2/M fraction to 56.4 ± 4.0% (*p* < 0.001), consistent with mitotic arrest-associated activity.

The TQ + Dos combination induced the highest degree of G2/M accumulation among all experimental groups, reaching 61.2 ± 4.4% (*p* < 0.001 versus control). Simultaneously, the S-phase population decreased markedly from 28.6 ± 2.4% in control cells to 9.1 ± 1.1% following combination treatment. In addition, a pronounced increase in the sub-G1 population was observed in the combination group (14.8 ± 1.6%), suggesting enhanced apoptotic DNA fragmentation.

These findings indicate that combined TQ and Dos exposure strongly disrupts cell cycle progression in OVCAR3 cells and is associated with marked accumulation of cells within the G2/M phase.

### 3.5. Modulation of Cytokine Profiles Following TQ and Dos Treatment

**Figure 7:**
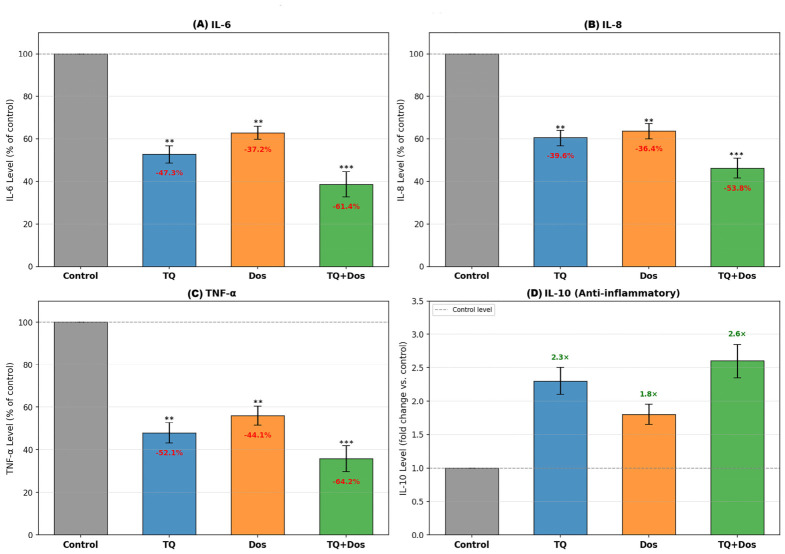
Effects of TQ, Dos, and the TQ + Dos combination on cytokine profiles in OVCAR3 cells determined by ELISA analysis. (**A**) Relative IL-6 levels following treatment exposure. TQ and Dos monotherapies reduced IL-6 production, whereas the combination treatment produced the greatest suppressive effect. (**B**) Relative IL-8 levels in treated OVCAR3 cells. Combination treatment induced the strongest reduction in IL-8 expression compared with monotherapy groups. (**C**) Relative TNF-α levels following treatment exposure. TQ + Dos treatment markedly suppressed TNF-α production relative to control cells. (**D**) Relative IL-10 expression levels. Treatment groups demonstrated increased IL-10 expression compared with control cells, with the highest induction observed in the combination group. Data are presented as mean ± SD (*n* = 3) from three independent biological experiments. Statistical significance was determined using one-way ANOVA followed by Tukey’s post hoc test (** *p* < 0.01, *** *p* < 0.001 versus control).

The effects of TQ, Dos, and combination treatment on inflammatory cytokine production in OVCAR3 cells were evaluated using ELISA-based analysis (Figure 7). Treatment-associated alterations were observed in both pro-inflammatory and anti-inflammatory cytokine profiles relative to the control group.

TQ treatment significantly reduced the levels of pro-inflammatory cytokines, including IL-6, IL-8, and TNF-α. Compared with control cells, IL-6 levels decreased by 47.3 ± 4.1%, IL-8 levels by 39.6 ± 3.7%, and TNF-α levels by 52.1 ± 4.8%. Dos treatment also reduced pro-inflammatory cytokine expression, although the suppressive effect was less pronounced than that observed with TQ treatment. In the Dos group, IL-6, IL-8, and TNF-α levels decreased by 37.2 ± 3.1%, 36.4 ± 3.6%, and 44.1 ± 4.4%, respectively.

The TQ + Dos combination produced the strongest suppression of pro-inflammatory cytokines among all treatment groups. Combination treatment reduced IL-6 levels by 61.4 ± 5.9%, IL-8 levels by 53.8 ± 4.7%, and TNF-α levels by 64.2 ± 6.1% relative to control cells (*p* < 0.001). In parallel with the reduction in pro-inflammatory mediators, anti-inflammatory IL-10 expression increased following treatment exposure. IL-10 levels increased approximately 2.3-fold in the TQ group, 1.8-fold in the Dos group, and 2.6-fold in the combination group compared with control cells.

These findings indicate that combined TQ and Dos treatment is associated with marked modulation of cytokine production in OVCAR3 cells, characterized by suppression of pro-inflammatory cytokines together with increased IL-10 expression.

### 3.6. Effects of TQ and Dos on Apoptosis-Related Gene Expression

**Figure 8:**
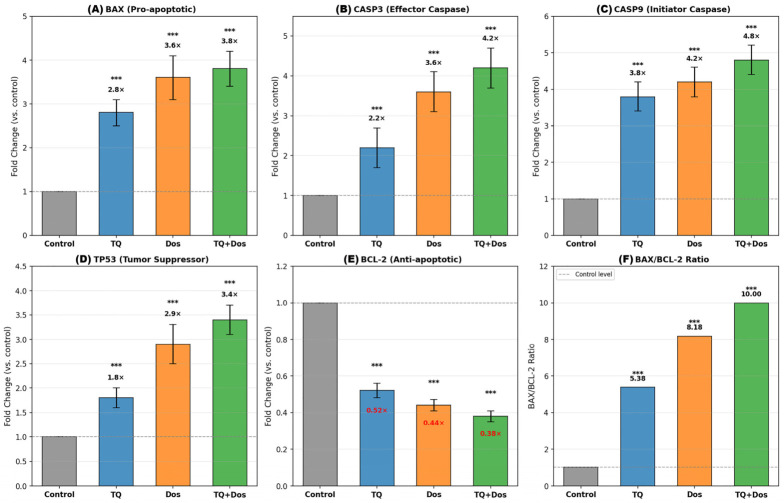
RT-qPCR analysis of apoptosis-associated gene expression in OVCAR3 cells following 48 h treatment with TQ, Dos, and the TQ + Dos combination. (**A**) RSelative expression levels of the pro-apoptotic gene *BAX*. (**B**) Relative expression of *CASP3*. (**C**) Relative expression of *CASP9*. (**D**) Relative expression levels of *TP53*. (**E**) Relative expression of the anti-apoptotic gene *BCL2*. (**F**) Quantitative analysis of the *BAX/BCL2* ratio. Treatment groups demonstrated increased expression of pro-apoptotic genes together with suppression of *BCL2* expression, whereas the TQ + Dos combination produced the most pronounced transcriptional alterations. Gene expression levels were normalized to the control group using the 2^−ΔΔCt^ method. Data are presented as mean ± SD (*n* = 3) from three independent biological experiments (*** *p* < 0.001 versus control).

The effects of TQ, Dos, and combination treatment on apoptosis-associated gene expression profiles were evaluated in OVCAR3 cells using RT-qPCR analysis following 48 h exposure (Figure 8). Treatment-induced alterations were observed in both pro-apoptotic and anti-apoptotic gene expression patterns.

Expression of the pro-apoptotic gene *BAX* increased significantly in all treatment groups compared with the control condition. *BAX* expression increased approximately 2.8-fold following TQ treatment, 3.6-fold following Dos treatment, and 3.8-fold following combination treatment (Figure 8A). Similarly, expression levels of the apoptotic caspase genes *CASP3* and *CASP9* were markedly elevated after treatment exposure. *CASP3* expression increased by approximately 2.2-fold in the TQ group, 3.6-fold in the Dos group, and 4.2-fold in the TQ + Dos group (Figure 8B). Expression of *CASP9* increased by approximately 3.8-fold, 4.2-fold, and 4.8-fold in the TQ, Dos, and combination groups, respectively (Figure 8C).

In parallel with pro-apoptotic activation, expression of the tumor suppressor gene *TP53* also increased following treatment exposure. Relative *TP53* expression levels increased approximately 1.8-fold in the TQ group, 2.9-fold in the Dos group, and 3.4-fold in the combination group compared with control cells (Figure 8D).

Conversely, the anti-apoptotic gene *BCL2* was significantly downregulated in all treatment groups (Figure 8E). *BCL2* expression decreased to approximately 0.52-fold of control levels following TQ treatment, 0.44-fold following Dos treatment, and 0.38-fold following combination treatment. Consistent with these findings, the *BAX/BCL2* ratio increased markedly after treatment exposure, reaching 5.38 in the TQ group, 8.18 in the Dos group, and 10.00 in the TQ + Dos group (Figure 8F).

Among all experimental conditions, the TQ + Dos combination produced the strongest modulation of apoptosis-associated gene expression, characterized by simultaneous upregulation of pro-apoptotic mediators and suppression of anti-apoptotic signaling.

**Figure 9:**
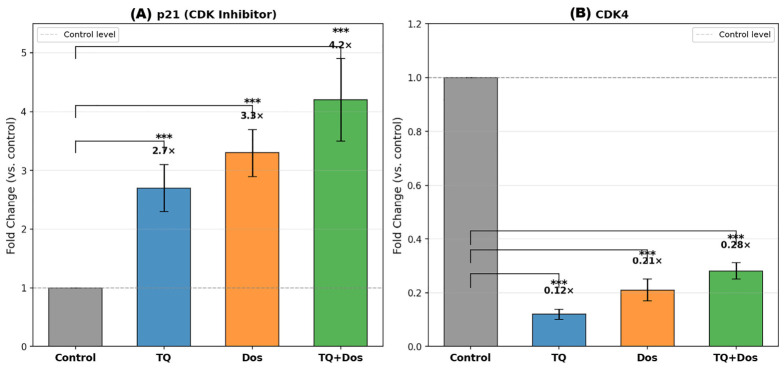
RT-qPCR analysis of cell cycle regulatory gene expression in OVCAR3 cells following 48 h treatment with TQ, Dos, and the TQ + Dos combination. (**A**) Relative expression levels of the cyclin-dependent kinase inhibitor *CDKN1A* (*p21*). Treatment groups demonstrated increased *p21* expression compared with control cells, with the strongest induction observed following combination treatment. (**B**) Relative expression levels of *CDK4*. All treatment groups exhibited reduced *CDK4* expression relative to the control group. Gene expression levels were normalized using the 2^−ΔΔCt^ method and expressed relative to control cells. Data are presented as mean ± SD (*n* = 3) from three independent biological experiments (*** *p* < 0.001 versus control).

To further investigate the molecular basis of treatment-associated cell cycle alterations, the expression levels of key cell cycle regulatory genes were evaluated in OVCAR3 cells using RT-qPCR analysis following 48 h treatment (Figure 9). Expression of the cyclin-dependent kinase inhibitor CDKN1A (p21) increased significantly in all treatment groups compared with control cells (Figure 9A). Relative p21 expression increased approximately 2.7-fold following TQ treatment, 3.3-fold following Dos treatment, and 4.2-fold following combination treatment. The highest induction was observed in the TQ + Dos group, consistent with the marked G2/M phase accumulation detected in flow cytometric analyses. In contrast, expression of CDK4, a key regulator of cell cycle progression, was substantially reduced following treatment exposure (Figure 9B). Relative CDK4 expression decreased to approximately 0.12-fold of control levels in the TQ group, 0.21-fold in the Dos group, and 0.28-fold in the combination group.

These findings demonstrate that TQ and Dos treatment modulates the transcriptional profile of genes associated with cell cycle regulation and supports the observed disruption of cell cycle progression in OVCAR3 cells.

### 3.7. Effects of Treatment on Active Caspase-3 Expression

**Figure 10:**
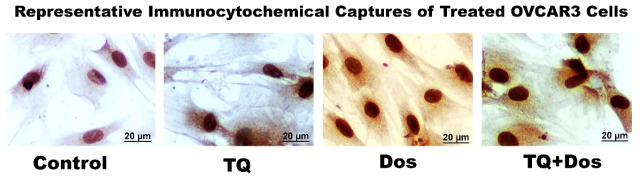
Representative immunocytochemical images of OVCAR3 cells following 48 h treatment with TQ, Dos, and the TQ + Dos combination. Active caspase-3 expression was visualized using DAB chromogen staining. Control cells displayed preserved morphology with weak basal staining, whereas treated groups demonstrated progressively increased cytoplasmic DAB reactivity and treatment-associated morphological alterations. The TQ + Dos group exhibited the strongest staining intensity together with marked cellular shrinkage and structural disruption. Scale bar: 20 µm. Images are representative of three independent biological experiments (*n* = 3).

**Figure 11:**
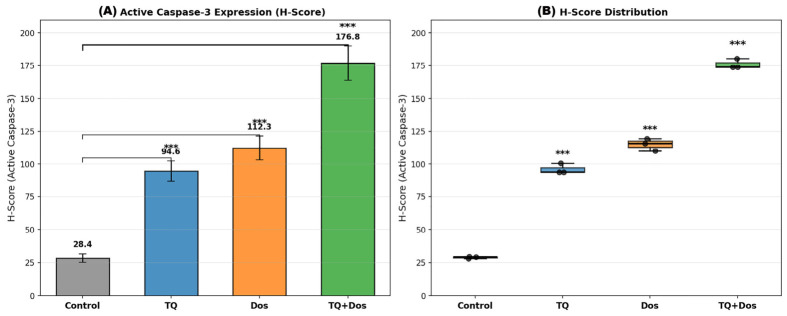
Immunocytochemical quantification of active caspase-3 expression in OVCAR3 cells following treatment with TQ, Dos, and the TQ + Dos combination. (**A**) Semi-quantitative H-score analysis of active caspase-3 immunoreactivity. Combination treatment produced the highest H-score values compared with monotherapy and control groups. (**B**) Distribution of H-score measurements presented as box plots with individual experimental values. Data are presented as mean ± SD (*n* = 3) from three independent biological experiments (*** *p* < 0.001 versus control).

Active caspase-3 immunoreactivity was evaluated in OVCAR3 cells following 48 h treatment with TQ, Dos, and the TQ + Dos combination using DAB-based immunocytochemical staining (Figure 10 and Figure 11). Distinct treatment-associated differences in staining intensity and cellular morphology were observed among experimental groups.

Control cells exhibited preserved cellular architecture with weak basal DAB staining and minimal active caspase-3 immunoreactivity (Figure 10). TQ-treated cells demonstrated moderate increases in cytoplasmic staining intensity together with mild morphological alterations. Dos treatment produced stronger active caspase-3 staining accompanied by more evident apoptotic morphology, including cellular shrinkage and irregular cytoplasmic organization.

The TQ + Dos combination generated the most intense staining pattern among all treatment groups. Combination-treated cells exhibited marked cytoplasmic DAB positivity together with pronounced structural disruption and condensed cellular morphology, consistent with enhanced apoptotic activity.

Semi-quantitative H-score analysis confirmed the microscopic observations (Figure 11). Active caspase-3 H-score values increased from 28.3 ± 2.5 in the control group to 94.7 ± 8.0 following TQ treatment and 112.3 ± 8.7 following Dos treatment. The highest immunoreactivity was detected in the TQ + Dos group, which reached an H-score value of 176.7 ± 14.1 (*p* < 0.001 versus control).

The elevated active caspase-3 staining observed in the combination group paralleled the apoptosis-associated alterations detected in flow cytometry and RT-qPCR analyses.

### 3.8. Fluorescence Visualization of Intracellular ROS Accumulation in OVCAR3 Cells

**Figure 12:**
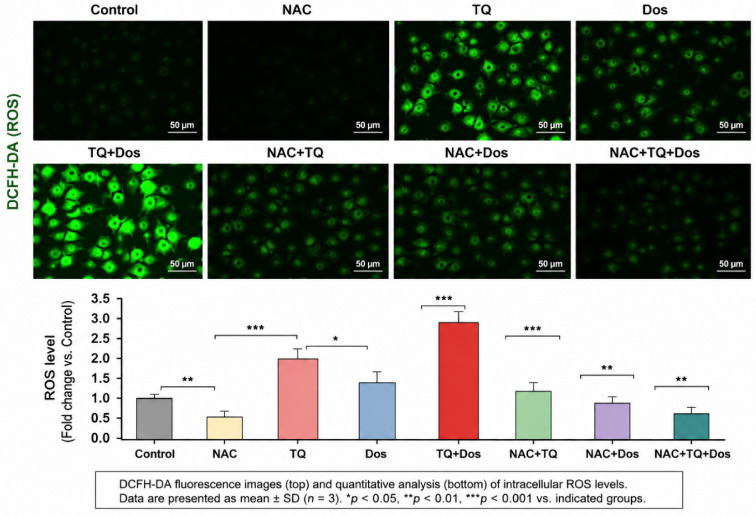
Fluorescence-based visualization and quantitative analysis of intracellular ROS accumulation in OVCAR3 cells following NAC, TQ, Dos, and combination treatments. Representative DCFH-DA fluorescence captures demonstrating intracellular ROS-associated green fluorescence intensity in control, NAC, TQ, Dos, TQ + Dos, NAC + TQ, NAC + Dos, and NAC + TQ + Dos treatment groups. Control and NAC-treated cells exhibited low basal fluorescence intensity, whereas TQ and Dos treatments increased intracellular ROS-associated fluorescence. The TQ + Dos combination produced the strongest fluorescence signal, indicating marked oxidative stress accumulation. NAC pretreatment reduced fluorescence intensity in treatment groups, particularly in the NAC + TQ + Dos group. Quantitative fluorescence analysis confirmed significant ROS elevation following TQ, Dos, and especially TQ + Dos treatment. Statistical comparisons indicated significant differences between selected experimental groups as presented in the figure (* *p* < 0.05, ** *p* < 0.01, *** *p* < 0.001). Scale bar: 50 µm. Representative images and quantitative data were obtained from three independent biological experiments (*n* = 3).

Representative DCFH-DA fluorescence captures demonstrated low basal intracellular ROS-associated fluorescence intensity in control and NAC-treated OVCAR3 cells, whereas TQ and Dos treatments increased intracellular fluorescence intensity (Figure 12). The TQ + Dos combination produced the strongest fluorescence signal together with marked fluorescence heterogeneity, indicating substantial intracellular ROS accumulation compared with single-agent treatment groups. NAC pretreatment attenuated fluorescence intensity in TQ-, Dos-, and particularly TQ + Dos-treated cells, although residual ROS-associated fluorescence remained detectable in the NAC + TQ + Dos group.

Quantitative fluorescence analysis further supported these observations (Figure 12). Compared with the control group, both TQ and Dos treatments increased intracellular ROS levels. Notably, the TQ + Dos combination resulted in the highest ROS accumulation (~2.9-fold vs. control; *p* < 0.001). NAC pretreatment significantly reduced ROS levels in TQ-, Dos-, and combination-treated groups, confirming effective suppression of treatment-associated oxidative stress. However, ROS levels in NAC-pretreated combination groups remained above basal control levels, suggesting partial rather than complete inhibition of oxidative stress-mediated responses.

Taken together, these fluorescence imaging and quantitative findings demonstrate that the TQ + Dos combination markedly enhances intracellular ROS accumulation in OVCAR3 cells, while NAC pretreatment partially attenuates treatment-associated oxidative stress (Figure 12).

### 3.9. Enhanced Antitumor Activity of the TQ–Dos Combination in 3D OVCAR3 Tumor Spheroids

**Figure 13:**
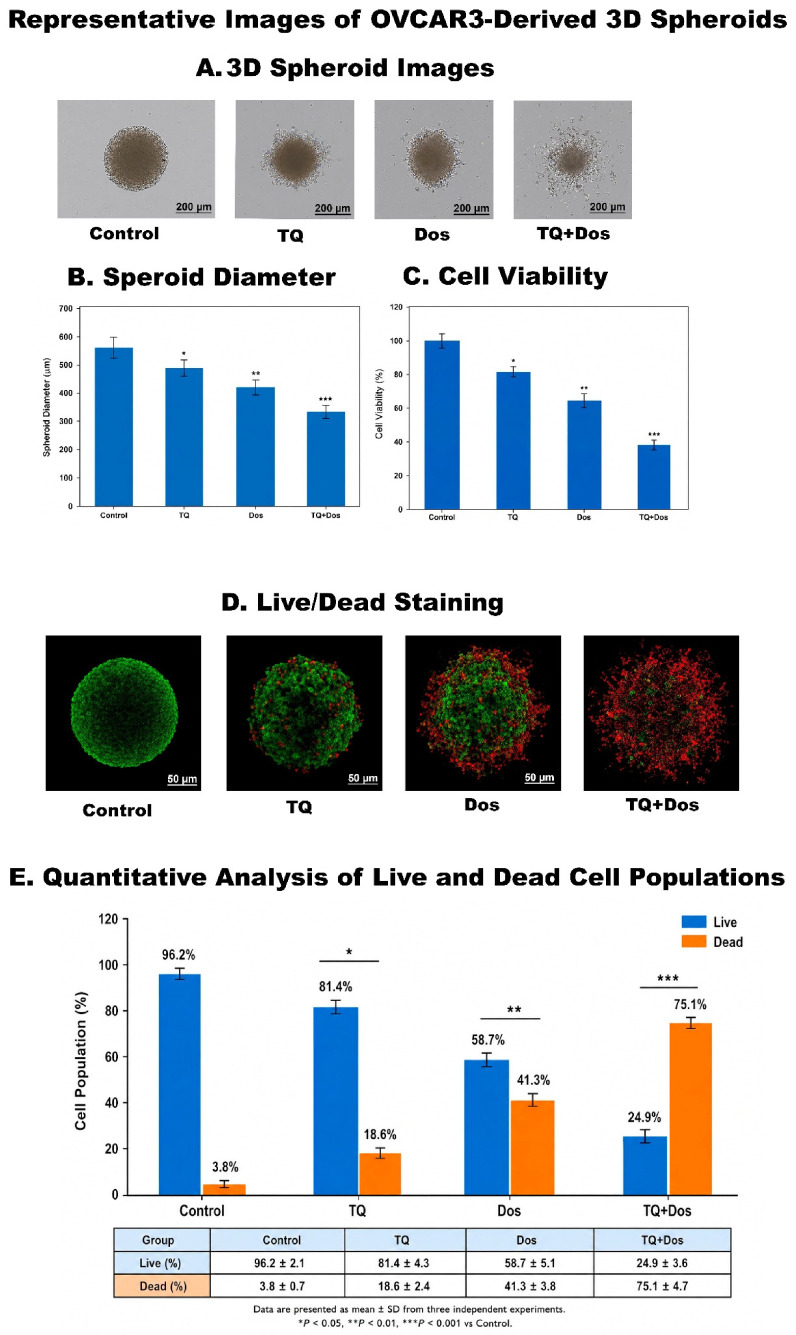
Enhanced antitumor effects of TQ and Dos combination treatment in OVCAR3-derived 3D tumor spheroids. (**A**) Representative bright-field captures of OVCAR3-derived 3D spheroids following treatment with control, TQ, Dos, or their combination (TQ + Dos). Control spheroids exhibited compact and well-organized morphology, whereas treated groups demonstrated progressive spheroid shrinkage, structural disruption, and loss of spheroid integrity, most prominently in the combination group. Scale bar: 200 µm. (**B**) Quantitative analysis of spheroid diameter showing significant reduction in spheroid size following TQ and Dos treatment, with the greatest decrease observed in the TQ + Dos group. (**C**) Quantitative analysis of spheroid cell viability demonstrating progressive reduction in viability across treatment groups, with the strongest inhibitory effect detected following combination treatment. (**D**) Representative live/dead fluorescence captures using Calcein-AM (green, viable cells) and Ethidium homodimer-1 (red, dead cells). Control spheroids displayed predominantly green fluorescence with preserved spheroid architecture, whereas treated spheroids exhibited progressively increased red fluorescence, cellular disorganization, and structural disruption, most markedly in the TQ + Dos group. Scale bar: 50 µm. (**E**) Quantitative analysis of live and dead cell populations demonstrating progressive loss of viable cells and increased dead-cell proportion following treatment, with the highest cytotoxic effect observed in the TQ + Dos group. Data are presented as mean ± SD (*n* = 3) from three independent biological experiments. Statistical analysis was performed using one-way ANOVA followed by Tukey’s post hoc test (* *p* < 0.05, ** *p* < 0.01, *** *p* < 0.001 vs. control).

Under control conditions, OVCAR3 cells formed compact, spherical, and structurally well-organized 3D spheroids with smooth borders and preserved architectural integrity. TQ treatment induced moderate morphological alterations characterized by mild peripheral loosening and partial reduction in spheroid compactness while largely maintaining overall spheroid organization. In contrast, Dos treatment produced more pronounced structural disruption, including irregular spheroid borders, decreased compactness, and increased peripheral cellular dispersion. Notably, the TQ + Dos combination resulted in the most extensive architectural deterioration, characterized by severe spheroid shrinkage, marked loss of structural integrity, widespread cellular scattering, and advanced fragmentation, consistent with substantial impairment of spheroid organization (Figure 13A).

Quantitative analysis of spheroid diameter supported these morphological observations (Figure 13B). Compared with control spheroids (~560 µm), TQ treatment moderately reduced spheroid diameter, whereas Dos treatment induced a more substantial decrease. Importantly, the TQ + Dos combination produced the greatest reduction in spheroid size (~320–350 µm), significantly lower than both single-agent treatment groups (*p* < 0.01–0.001), indicating enhanced growth-inhibitory activity under 3D culture conditions.

ATP-based viability analysis further demonstrated that both TQ and Dos monotherapies significantly reduced spheroid viability relative to control spheroids (Figure 13C). TQ treatment reduced viability to approximately 80–85%, whereas Dos treatment decreased viability to nearly 60–65%. Notably, the combination group exhibited the strongest cytotoxic response, reducing viability to approximately 35–40% of control levels (*p* < 0.001 vs. single-agent groups), consistent with maintenance of the stronger cytotoxic response observed with combination treatment in the 3D ovarian cancer model.

Live/dead fluorescence imaging further confirmed treatment-associated cytotoxicity (Figure 13D). Control spheroids displayed intense and homogeneous green fluorescence with minimal red signal, indicating high viability and preserved spheroid architecture. TQ-treated spheroids exhibited a mild increase in red fluorescence accompanied by limited peripheral disorganization, consistent with moderate induction of cell death. Dos-treated spheroids demonstrated substantially increased red fluorescence together with more evident structural disruption and reduced compactness. In contrast, TQ + Dos-treated spheroids exhibited dominant red fluorescence, marked reduction in green signal intensity, severe spheroid fragmentation, and extensive structural collapse, indicating widespread loss of viability and profound disruption of spheroid organization.

Quantitative analysis of live and dead cell populations paralleled fluorescence imaging findings (Figure 13E). Control spheroids contained predominantly viable cells (~96% live cells), whereas TQ and Dos treatments progressively increased the proportion of non-viable cells. The TQ + Dos combination produced the highest dead-cell fraction (~75%) together with the lowest proportion of viable cells (~25%), supporting the marked cytotoxic effect observed microscopically. Because fluorescence signal intensity and spatial distribution may vary within dense spheroid structures, live/dead imaging results were interpreted qualitatively alongside ATP-based viability analyses.

Together, these findings indicate that the stronger cytotoxic and growth-inhibitory effects observed in monolayer cultures are also observed in a 3D ovarian cancer model and are associated with increased cytotoxic and growth-inhibitory effects under diffusion-limited tumor-like conditions rather than definitive evidence of a specific molecular mechanism.

### 3.10. ROS Contributes to, but Does Not Fully Mediate, TQ–Dos-Induced Cytotoxicity in 3D OVCAR3 Spheroids

**Figure 14:**
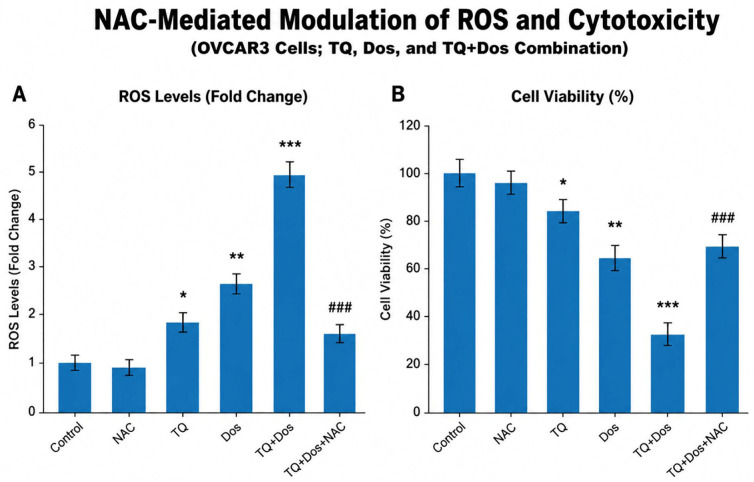
NAC-mediated modulation of ROS generation and cytotoxicity in OVCAR3 cells following TQ and Dos treatment. (**A**) Quantitative analysis of intracellular ROS levels measured by DCFH-DA fluorescence following treatment with NAC, TQ, Dos, or the TQ + Dos combination. Combination treatment markedly increased ROS production compared with single-agent treatments, whereas NAC pretreatment attenuated ROS accumulation induced by TQ + Dos. (**B**) Quantitative analysis of cell viability following treatment with NAC, TQ, Dos, or the TQ + Dos combination. TQ + Dos treatment significantly reduced cell viability, while NAC pretreatment partially restored cell survival, suggesting a role for ROS-mediated cytotoxicity in the observed antitumor effects. Data are presented as mean ± SD (*n* = 3) from three independent biological experiments. Statistical analysis was performed using one-way ANOVA followed by Tukey’s post hoc test (* *p* < 0.05, ** *p* < 0.01, *** *p* < 0.001 vs. control; ### *p* < 0.001 vs. TQ + Dos group).

To evaluate the functional contribution of oxidative stress to treatment-associated cytotoxicity, intracellular ROS levels and spheroid viability were assessed following NAC pretreatment (Figure 14A,B).

ROS analysis demonstrated low basal oxidative stress levels in control and NAC-only groups (Figure 14A). TQ treatment moderately increased intracellular ROS accumulation (~1.8-fold), whereas Dos treatment induced a more pronounced elevation (~2.6-fold). Notably, the TQ + Dos combination produced the highest ROS accumulation (~5.0-fold vs. control; *p* < 0.001), indicating enhanced oxidative stress under combination-treatment conditions. Pretreatment with NAC markedly attenuated ROS levels in the TQ + Dos group, reducing ROS accumulation to approximately 1.6-fold above control levels (### *p* < 0.001 vs. TQ + Dos), confirming effective suppression of oxidative stress.

Consistent with ROS modulation, viability analysis demonstrated that control and NAC-only spheroids maintained high viability, whereas TQ and Dos treatments reduced spheroid viability to approximately 84% and 64%, respectively (Figure 14B). The TQ + Dos combination produced the strongest cytotoxic effect, reducing viability to nearly 32% of control levels (*p* < 0.001). Importantly, NAC pretreatment partially restored spheroid viability in the combination group to approximately 68–70% (### *p* < 0.001 vs. TQ + Dos alone), indicating a functional rescue effect.

However, viability in NAC-pretreated spheroids remained substantially lower than untreated control levels, demonstrating that ROS scavenging only partially reverses treatment-associated cytotoxicity. These findings indicate that ROS generation functionally contributes to the cytotoxic effects associated with the TQ–Dos combination in 3D ovarian cancer spheroids but does not fully account for the observed antitumor activity. Accordingly, the data suggest the involvement of additional mechanisms, potentially including apoptosis-associated signaling and cell cycle-related responses, rather than ROS acting as the sole mediator of treatment-induced cytotoxicity.

### 3.11. Bioinformatics Analysis Findings

#### 3.11.1. PPI Network Analysis

**Figure 15:**
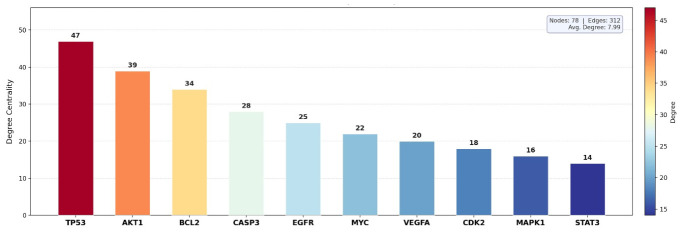
Degree centrality analysis of the top 10 hub genes identified from the protein–protein interaction (PPI) network associated with TQ + Dos treatment. The interaction network was constructed using the STRING database (v11.5; confidence score ≥ 0.700) and analyzed in Cytoscape (v3.10.0). The network consisted of 78 nodes and 312 interaction edges. *TP53* exhibited the highest degree centrality (degree = 47), followed by *AKT1* (degree = 39) and *BCL2* (degree = 34), indicating prominent connectivity within the interaction network. Additional highly connected hub genes included *CASP3*, *EGFR*, *MYC*, *VEGFA*, *CDK2*, *MAPK1*, and *STAT3*. This figure represents a computational bioinformatics analysis and does not involve biological replicates.

To explore potential molecular interactions associated with the TQ + Dos treatment network, a protein–protein interaction (PPI) network was constructed using the STRING database and further analyzed in Cytoscape (Figure 15). The generated interaction network consisted of 78 nodes and 312 interaction edges, indicating extensive connectivity among the identified target proteins.

Topological analysis identified the top 10 hub genes according to degree centrality values. *TP53* was identified as the dominant hub node with the highest degree value (degree = 47), followed by *AKT1* (degree = 39) and *BCL2* (degree = 34). Additional highly connected genes included *CASP3*, *EGFR*, *MYC*, *VEGFA*, *CDK2*, *MAPK1*, and *STAT3*.

The prominence of apoptosis-related and survival-associated hub genes within the network suggests that the biological activity of the TQ + Dos combination may involve coordinated regulation of apoptosis signaling, cell survival pathways, and proliferative signaling networks. In particular, the central positioning of *TP53*, *BCL2*, and *CASP3* within the interaction map was consistent with the experimentally observed apoptosis-associated molecular alterations identified in RT-qPCR and immunocytochemical analyses.

#### 3.11.2. GO and KEGG Enrichment Analysis

Functional enrichment analyses were performed to investigate the biological relevance of the overlapping target genes associated with TQ and Dos treatment. GO enrichment analysis demonstrated significant association of the identified targets with apoptosis-related and cell cycle-associated biological processes. Among the most significantly enriched GO categories were apoptotic process (GO:0006915), cell cycle regulation (GO:0051726), protein kinase activity (GO:0004672), and transcription factor binding (GO:0008134) (FDR < 0.001).

KEGG pathway enrichment analysis identified several significantly enriched signaling pathways associated with cellular survival, apoptosis, and proliferation. The most enriched pathways included PI3K–AKT signaling (hsa04151; *p* = 2.3 × 10^−8^), p53 signaling (hsa04115; *p* = 4.7 × 10^−7^), apoptosis (hsa04210; *p* = 8.1 × 10^−7^), and cell cycle regulation (hsa04110; *p* = 1.2 × 10^−6^).

The enrichment profile obtained from computational analysis was compatible with the experimentally observed alterations in apoptosis-related gene expression and cell cycle progression identified in OVCAR3 cells following TQ and Dos treatment.

## 4. Discussion

In the present study, the combined effects of TQ and Dos were comprehensively evaluated in OVCAR3 ovarian cancer cells using integrated cellular, molecular, fluorescence-based, three-dimensional spheroid, and bioinformatic approaches. The findings demonstrated that combined treatment produced stronger cytotoxic, apoptotic, and antiproliferative effects than either monotherapy alone, accompanied by marked alterations in apoptosis-associated gene expression, cell cycle progression, intracellular ROS levels, and cytokine profiles. MTT and combination index analyses revealed enhanced cytotoxic activity of the TQ + Dos combination together with synergistic interaction patterns in OVCAR3 cells, while relatively lower toxicity was observed in HaCaT keratinocyte cells. In parallel with these findings, Annexin V-FITC/PI analyses demonstrated increased apoptotic cell populations following combination treatment, whereas RT-qPCR and immunocytochemical analyses showed increased expression of pro-apoptotic mediators including *BAX*, *CASP3*, *CASP9*, and *TP53*, accompanied by suppression of *BCL2* expression and dysregulation of cell cycle-associated genes. Fluorescence-based DCFH-DA imaging together with NAC pretreatment experiments further suggested that oxidative stress modulation may contribute, at least partially, to the enhanced cytotoxic activity observed following combined exposure. Importantly, 3D spheroid experiments demonstrated reduced spheroid integrity, decreased viability, and increased dead-cell accumulation in the TQ + Dos group, supporting the biological activity of the combination under more physiologically relevant culture conditions. Previous studies have reported that TQ exhibits antitumor activity in ovarian cancer models through modulation of apoptosis-associated and survival-related signaling pathways [19,20,21,22]. However, the present study expands these observations by integrating ROS-associated cellular stress responses, NAC-mediated modulation experiments, and 3D spheroid validation into the evaluation of combined TQ and Dos treatment. Together, the findings suggest that combined TQ and Dos exposure exerts multifactorial antitumor effects in OVCAR3 cells associated with apoptosis-related signaling, disruption of cell cycle progression, inflammatory cytokine modulation, and ROS-associated cellular stress responses.

MTT analysis demonstrated that TQ reduced OVCAR3 cell viability in a dose- and time-dependent manner, with an IC_50_ value of 85.4 µM after 48 h of exposure. These findings are generally consistent with previous reports demonstrating IC_50_ values ranging between 50–120 µM in different ovarian cancer cell lines [19,20]. Kaseb et al. reported that TQ suppresses proliferation and modulates survival-associated signaling pathways, including PI3K/AKT-related mechanisms, in cancer cell models [21]. In the present study, the relatively higher IC_50_ values observed in HaCaT cells suggest that TQ-associated cytotoxicity may preferentially affect ovarian cancer cells compared with non-tumoral keratinocytes, which is compatible with previous observations reported by Gali-Muhtasib et al. [22]. Nevertheless, measurable cytotoxic responses were also observed in HaCaT cells, indicating that the selectivity profile of the TQ + Dos combination should be interpreted cautiously and requires further validation under in vivo conditions.

Dos treatment produced marked cytotoxicity at nanomolar concentrations in OVCAR3 cells, which is consistent with the established antitumor activity of taxane-based agents in ovarian cancer treatment [23]. Importantly, combination treatment with TQ and Dos generated stronger suppression of cell viability than either monotherapy alone. CI analyses performed according to the Chou–Talalay method demonstrated CI values below 1 across all evaluated effect levels in OVCAR3 cells, supporting a synergistic interaction between the two agents [24]. Similar synergistic interactions between TQ and conventional chemotherapeutic agents including doxorubicin [25], methotrexate [11], and gemcitabine [26] have previously been reported in different cancer models. The current findings extend these observations to OVCAR3 ovarian cancer cells and further support the enhanced biological activity of combined TQ and Dos exposure.

Flow cytometric analyses demonstrated that the TQ + Dos combination markedly increased apoptotic cell populations compared with either monotherapy alone. Combination treatment produced substantial expansion of both early and late apoptotic fractions, consistent with enhanced apoptosis-associated cellular responses. In parallel with the flow cytometric findings, RT-qPCR analyses revealed increased expression of pro-apoptotic genes including *BAX*, *CASP3*, and *CASP9*, together with suppression of anti-apoptotic *BCL2* expression. The marked increase in the *BAX/BCL2* ratio observed following combination treatment further supports activation of apoptosis-associated signaling pathways. Previous studies demonstrated that TQ may disrupt mitochondrial homeostasis and contribute to caspase-associated apoptotic signaling in ovarian and other cancer models [27,28,29]. The balance between pro- and anti-apoptotic members of the BCL-2 family is known to play a critical role in the regulation of cancer cell survival and apoptotic susceptibility [30]. In the present study, immunocytochemical analyses demonstrated increased active caspase-3 immunoreactivity following combined treatment, which paralleled the transcriptional alterations observed by RT-qPCR analysis. Although these findings support apoptosis-associated molecular responses, the absence of comprehensive protein-level analyses such as Western blotting limits definitive mechanistic interpretation. Previous molecular docking studies additionally suggested that TQ may interact with the BCL-2 BH3 domain [12,31], which may partly explain the observed reduction in *BCL2* expression following treatment exposure.

Cell cycle analyses demonstrated that the TQ + Dos combination markedly enhanced G2/M phase accumulation in OVCAR3 cells. Consistent with the established mechanism of taxane-based agents, Dos treatment produced pronounced G2/M arrest through disruption of mitotic progression [32]. In the present study, combination treatment further intensified G2/M accumulation and was accompanied by increased expression of *CDKN1A* (*p21*) together with suppression of *CDK4* expression. The observed increase in *TP53* expression may also be associated with altered cell cycle checkpoint regulation [33]. However, because direct pathway-specific analyses were not performed, these transcriptional alterations should be interpreted as supportive molecular associations rather than definitive evidence of p53-dependent signaling activation. Previous studies have also suggested that suppression of CDC25C-associated signaling may contribute to mitotic checkpoint dysregulation and G2/M arrest mechanisms [34].

An important aspect of the present study was the incorporation of fluorescence-based ROS analyses together with NAC pretreatment experiments. DCFH-DA fluorescence imaging demonstrated increased intracellular ROS accumulation following TQ and Dos treatment, with the strongest fluorescence intensity observed in the combination group. NAC pretreatment partially attenuated ROS accumulation and was associated with partial recovery of cell viability, suggesting that oxidative stress modulation may contribute to the cytotoxic effects induced by combined treatment. However, because NAC treatment did not completely reverse treatment-associated viability loss, the observed biological effects are unlikely to be explained solely by ROS-associated mechanisms. Instead, the findings suggest that oxidative stress modulation may act together with apoptosis-associated signaling and cell cycle disruption as part of a multifactorial response pattern. These observations were further supported by increased apoptotic cell accumulation, elevated *BAX/BCL2* ratios, and enhanced active caspase-3 immunoreactivity detected following combined treatment.

The 3D spheroid experiments provided additional support for the biological activity of combined TQ and Dos treatment under more physiologically relevant conditions. Compared with monotherapy groups, the TQ + Dos combination induced more pronounced spheroid shrinkage, reduced ATP-based viability, and increased dead-cell accumulation in live/dead fluorescence analyses. Since 3D spheroid systems more closely mimic tumor architecture, nutrient gradients, and diffusion-associated treatment limitations than conventional monolayer cultures, these findings strengthen the translational relevance of the observed antitumor responses. Nevertheless, although 3D spheroid systems represent a more advanced experimental platform compared with 2D cultures, they still cannot fully reproduce the complexity of the in vivo tumor microenvironment. Recent advances in ovarian cancer management have increasingly emphasized molecular stratification, particularly BRCA1/2 mutation status and HRD, which are important determinants of responsiveness to PARP inhibitor-based therapies. Although the present study was not designed to evaluate BRCA- or HRD-associated mechanisms, the observed cytotoxic, apoptosis-associated, and spheroid-level responses following TQ + Dos treatment suggest that this combination may warrant future investigation in molecularly characterized ovarian cancer models. Future studies incorporating BRCA-mutated, HRD-positive, and PARP inhibitor-resistant ovarian cancer systems may help clarify the potential relevance of this combination within contemporary ovarian cancer treatment strategies. Beyond their utility for evaluating treatment efficacy, advanced three-dimensional tumor models are increasingly being recognized as valuable platforms for the preclinical assessment of emerging immunotherapeutic strategies. Recent advances in cancer vaccine development, particularly nanotechnology-enabled vaccine platforms, have highlighted the importance of physiologically relevant tumor models for evaluating treatment penetration, tumor-cell responses, and microenvironment-associated therapeutic effects. Although vaccine-related mechanisms were not investigated in the present study, the pronounced antitumor activity observed in the OVCAR3 spheroid model may support future studies exploring the integration of combination therapies with emerging immunotherapeutic approaches, including cancer nanovaccines and other next-generation vaccine platforms [35,36].

The TQ + Dos combination also significantly suppressed pro-inflammatory cytokines including IL-6, IL-8, and TNF-α while increasing IL-10 levels in OVCAR3 cells. IL-6 and IL-8 are known to contribute to ovarian cancer progression, angiogenesis, and treatment resistance [37,38]. Previous studies suggested that TQ may suppress inflammatory cytokine production partly through modulation of NF-κB-associated signaling pathways [39]. In the present study, the strongest cytokine modulation was observed following combination treatment. However, because cytokine measurements were performed using tumor-cell culture supernatants in the absence of immune-cell components, the immunological implications of these findings should be interpreted cautiously. Similarly, although CIBERSORT-associated bioinformatic analyses suggested potential interactions with immune-related pathways [40], these computational predictions require further experimental validation using immune-competent systems.

Bioinformatic analyses further supported the experimentally observed molecular alterations by identifying apoptosis- and survival-associated genes among the central nodes of the interaction network. PPI analysis identified *TP53*, *AKT1*, and *BCL2* as major hub genes, whereas GO and KEGG enrichment analyses demonstrated significant associations with apoptosis, cell cycle regulation, and PI3K–AKT signaling pathways. Since the PI3K/AKT pathway is frequently associated with ovarian cancer progression and taxane resistance [41], the enrichment profile identified in the current study may support the biological relevance of the experimentally observed treatment responses. Previous molecular docking studies also suggested potential interaction of TQ with AKT1-associated binding regions [42]. Nevertheless, the bioinformatic findings presented here should be interpreted as hypothesis-generating computational associations rather than direct evidence of pathway inhibition or activation.

The relatively lower apoptosis rates, higher IC_50_ values, and more moderate molecular alterations observed in HaCaT cells suggest partial selectivity of the TQ + Dos combination toward ovarian cancer cells. This difference may partly reflect altered oxidative stress responses, defective DNA repair mechanisms, and mitotic checkpoint abnormalities commonly observed in malignant cells [43,44,45]. However, because combination treatment also produced measurable cytotoxicity in HaCaT cells, comprehensive in vivo toxicity evaluation will be necessary before considering translational applications.

Previous studies demonstrated that TQ may exert antitumor activity in ovarian cancer cells through coordinated modulation of apoptosis-associated signaling pathways, oxidative stress responses, and mitochondrial dysfunction [46,47]. Taha et al. [46] reported that TQ induced ROS accumulation together with alterations in mitochondrial membrane permeability and apoptosis-associated molecular responses in ovarian cancer cells. Similarly, Karaosmanoğlu et al. [47] demonstrated increased *TP53* and *CASP3* expression accompanied by reduced OVCAR3 cell viability following TQ exposure. In parallel with these findings, the present study demonstrated that combined TQ and Dos treatment markedly increased apoptotic cell accumulation together with elevated expression of *BAX*, *CASP3*, *CASP9*, and *TP53*; suppression of *BCL2* expression; and increased active caspase-3 immunoreactivity. The increase in the *BAX/BCL2* ratio together with enhanced apoptotic cell accumulation observed following combination treatment supports activation of apoptosis-associated molecular responses under combined exposure conditions.

Previous reviews further suggested that the anticancer activity of TQ may involve multiple interconnected biological processes including apoptosis induction, ROS production, cell cycle arrest, inflammatory signaling modulation, and regulation of oncogenic signaling pathways such as AKT, *STAT3*, *ERK1/2*, and *NF-κB* [48,49]. In the present study, combined TQ and Dos treatment produced marked G2/M phase accumulation together with increased *CDKN1A (p21)* expression and suppression of *CDK4* expression, findings that are generally compatible with previously reported multifunctional effects of TQ [48,49]. Similarly, cytokine profiling analyses demonstrated significant suppression of *IL-6*, *IL-8*, and *TNF-α* levels together with increased *IL-10* expression following combination treatment, which may be associated with the previously described anti-inflammatory properties of TQ [48,49]. However, because pathway-specific activation assays were not performed in the current study, these molecular and transcriptional alterations should be interpreted as supportive mechanistic associations rather than definitive evidence of direct pathway inhibition or activation.

An important aspect of the present study was the incorporation of fluorescence-based ROS analyses together with NAC pretreatment experiments. DCFH-DA fluorescence imaging demonstrated increased intracellular ROS accumulation following combined TQ and Dos exposure, whereas NAC pretreatment partially attenuated ROS intensity and partially restored cell viability. Similar oxidative stress-associated responses were previously described by Taha et al. [46], who reported increased ROS accumulation and mitochondrial dysfunction following TQ treatment in ovarian cancer cells. Nevertheless, because NAC treatment in the present study did not completely reverse treatment-associated cytotoxicity, ROS-associated cellular stress alone is unlikely to fully explain the biological activity of the TQ + Dos combination. Instead, the findings support a multifactorial response pattern involving simultaneous modulation of oxidative stress responses, apoptosis-associated signaling, and cell cycle dysregulation.

The incorporation of 3D spheroid experiments further strengthened the biological relevance of the present findings. Compared with monotherapy groups, the TQ + Dos combination produced marked spheroid shrinkage together with reduced ATP-based viability and increased dead-cell accumulation in live/dead fluorescence analyses. Three-dimensional spheroid systems are increasingly recognized as more physiologically relevant experimental platforms because they better reproduce tumor architecture, cellular heterogeneity, nutrient and oxygen gradients, and treatment penetration characteristics compared with conventional monolayer cultures [50,51,52]. Flörkemeier et al. [50] demonstrated that multicellular ovarian cancer spheroids more effectively reproduce tumor microenvironment-associated interactions and treatment-associated biological responses than conventional monoculture systems. Similarly, Zhu et al. [51] and Arora et al. [52] emphasized that spheroid and organoid platforms provide improved representation of extracellular microenvironmental interactions, drug penetration behavior, soluble mediator gradients, and tumor-associated biological responses relative to oversimplified 2D systems. Previous studies comparing monolayer and spheroid cultures additionally demonstrated that 3D systems may exhibit altered sensitivity profiles to chemotherapeutic agents including docetaxel, highlighting the importance of incorporating spheroid-based platforms into preclinical drug evaluation studies [53]. In this context, the reduced spheroid viability and increased dead-cell accumulation observed following combined TQ and Dos treatment support the biological activity of the combination under more complex tumor-like culture conditions.

The present study has several limitations. First, experiments were conducted using a single ovarian cancer cell line, which may limit the generalizability of the findings across different ovarian cancer subtypes. Second, although fluorescence imaging, NAC modulation experiments, RT-qPCR, and active caspase-3 immunocytochemistry provided supportive mechanistic insight, comprehensive protein-level validation of the observed transcriptional alterations (including *BAX*, *BCL2*, *CASP9*, *TP53*, *CDKN1A*, and *CDK4*) was not performed. Therefore, these molecular findings should be interpreted as transcriptional associations rather than definitive evidence of corresponding protein-level regulation. Third, NAC treatment produced only partial attenuation of ROS accumulation and viability loss, suggesting that additional mechanisms likely contribute to the observed cytotoxic responses. Finally, the biological activity identified in 3D spheroid systems and bioinformatic analyses requires further validation in in vivo ovarian cancer models. Future studies incorporating multiple ovarian cancer cell lines, xenograft systems, transcriptomic analyses, and detailed pathway validation approaches may help clarify the therapeutic relevance of combined TQ and Dos treatment in ovarian cancer. Furthermore, HaCaT keratinocytes were used as a non-tumoral reference cell line to provide a preliminary assessment of treatment-associated cytotoxicity in non-malignant human cells. However, HaCaT cells do not represent normal ovarian epithelium. Therefore, the observed differences between OVCAR3 and HaCaT cells should not be interpreted as definitive evidence of ovarian cancer-specific selectivity, and future studies incorporating non-tumoral ovarian epithelial models are warranted.

## 5. Conclusions

The present study demonstrated that the combination of TQ and Dos produced greater cytotoxic, apoptotic, and growth-inhibitory effects in OVCAR3 ovarian cancer cells than either monotherapy alone. Combined treatment was associated with increased apoptotic cell accumulation, enhanced G2/M phase arrest, alterations in apoptosis- and cell cycle-associated gene expression, suppression of pro-inflammatory cytokines, increased intracellular ROS accumulation, and reduced spheroid viability under both 2D and 3D culture conditions. The concordance observed across flow cytometry, RT-qPCR, immunocytochemistry, ROS imaging, NAC rescue experiments, cytokine profiling, and spheroid analyses supports the consistency of the observed biological responses.

An important observation of the study was that NAC pretreatment only partially attenuated ROS accumulation and treatment-associated cytotoxicity, indicating that oxidative stress may contribute to, but does not solely account for, the biological effects of the TQ + Dos combination. Instead, the findings suggest that the observed responses may involve the simultaneous modulation of multiple apoptosis-, cell cycle-, and oxidative stress-associated processes rather than a single dominant mechanism.

Importantly, the biological effects observed following combined treatment were also evident in 3D ovarian cancer spheroids. The reductions in spheroid integrity and ATP-based viability together with increased dead-cell accumulation suggest that the activity of the combination is maintained under more complex tumor-like experimental conditions.

Overall, these in vitro findings suggest that the TQ + Dos combination may represent a promising strategy for further investigation in ovarian cancer models. The observed biological responses were associated with alterations in apoptosis-related signaling, oxidative stress responses, and cell cycle regulation. However, additional studies incorporating protein-level validation, pathway-specific analyses, and in vivo models will be necessary to further define the mechanistic and translational relevance of this combination.

## Figures and Tables

**Table 1:** Primer sequences.

Genes	Forward	Reverse
*CASP3*	GGAAGCGAATCAATGGACTCTGG	GCATCGACATCTGTACCAGACC
*CASP9*	GTTTGAGGACCTTCGACCAGCT	CAACGTACCAGGAGCCACTCTT
*BAX*	TCAGGATGCGTCCACCAAGAAG	TGTGTCCACGGCGGCAATCATC
*BCL-2*	ATCGCCCTGTGGATGACTGAGT	GCCAGGAGAAATCAAACAGAGGC
*TP53*	CCTCAGCATCTTATCCGAGTGG	TGGATGGTGGTACAGTCAGAGC
*p21*	AGGTGGACCTGGAGACTCTCAG	TCCTCTTGGAGAAGATCAGCCG
*CDK4*	CCATCAGCACAGTTCGTGAGGT	TCAGTTCGGGATGTGGCACAGA
*ACTB* (*β-Actin*)	CATTGCTGACAGGATGCAGAAGG	TGCTGGAAGGTGGACAGTGAGG
*GAPDH*	GGAGCGAGATCCCTCCAAAAT	GGCTGTTGTCATACTTCTCATGG

## Data Availability

The original contributions presented in this study are included in the article. Further inquiries can be directed at the corresponding authors.

## Abbreviations

AKT	Protein Kinase B
ANOVA	Analysis of Variance
ATP	Adenosine Triphosphate
BAX	BCL2 Associated X Protein
BCL2	B-Cell Lymphoma 2
BH3	BCL2 Homology 3
CASP3	Caspase-3
CASP9	Caspase-9
CDK4	Cyclin-Dependent Kinase 4
CI	Combination Index
DAB	3,3′-Diaminobenzidine
DCFH-DA	2′,7′-Dichlorodihydrofluorescein Diacetate
DMSO	Dimethyl Sulfoxide
Dos	Docetaxel
DRI	Dose Reduction Index
ELISA	Enzyme-Linked Immunosorbent Assay
ERK1/2	Extracellular Signal-Regulated Kinase 1/2
Fa	Fraction Affected
FBS	Fetal Bovine Serum
FDR	False Discovery Rate
FITC	Fluorescein Isothiocyanate
GO	Gene Ontology
H_2_O_2_	Hydrogen Peroxide
HRD	Homologous Recombination Deficiency
HRP	Horseradish Peroxidase
ICC	Immunocytochemistry
IC_50_	Half-Maximal Inhibitory Concentration
IL	Interleukin
KEGG	Kyoto Encyclopedia of Genes and Genomes
NAC	N-Acetylcysteine
NF-κB	Nuclear Factor Kappa B
PBS	Phosphate-Buffered Saline
PI	Propidium Iodide
PI3K	Phosphoinositide 3-Kinase
PPI	Protein–Protein Interaction
PARP	Poly Polymerase
qRT-PCR	Quantitative Real-Time Polymerase Chain Reaction
RNA	Ribonucleic Acid
ROS	Reactive Oxygen Species
RT-qPCR	Reverse Transcription Quantitative Polymerase Chain Reaction
SD	Standard Deviation
SEM	Standard Error of the Mean
STAT3	Signal Transducer and Activator of Transcription 3
STRING	Search Tool for the Retrieval of Interacting Genes/Proteins
TME	Tumor Microenvironment
TNF-α	Tumor Necrosis Factor-Alpha
TP53	Tumor Protein p53
TQ	Thymoquinone
VEGFA	Vascular Endothelial Growth Factor A
